# Effectiveness of perches in promoting bird-mediated seed dispersal for natural forest regeneration: a systematic review

**DOI:** 10.1186/s13750-025-00363-8

**Published:** 2025-06-14

**Authors:** Jelaine L. Gan, Matthew J. Grainger, Mark David Foster Shirley, Sheena Davis, Molly Watson, Shreya Dube, Marion Pfeifer

**Affiliations:** 1 School of Natural and Environmental Sciences, Newcastle University, Newcastle upon Tyne, UK; 2 Institute of Biology, College of Science, University of the Philippines Diliman, Quezon City, Philippines; 3 Norwegian Institute for Nature Research, Torgarden No. 7485, P.O. Box 5685, Trondheim, Norway; 4 School of Psychology and Neuroscience, University of St Andrews, St Mary’s Quad, South St, St Andrews, UK

**Keywords:** Reforestation, Regrowth, Avian, Frugivores, Artificial perch, Assisted natural regeneration, Nucleation, Meta-analysis

## Abstract

**Background:**

Assisted Natural Regeneration (ANR) is an increasingly popular cost-effective approach to restore forests for climate change mitigation and biodiversity conservation. One ANR strategy is the use of perches to attract avian seed dispersers to degraded landscapes for increased seed supply and seedling establishment. This systematic review sought to determine the effectiveness of artificial, semi-natural, and natural perches in promoting natural forest regeneration, specifically in driving four outcomes: seed richness, seed density, seedling richness, and seedling density.

**Methods:**

In September 2023, we searched for studies in eight bibliographic sources, which include an organizational library and a web-based search engine, using a refined search string in English. After deduplication, we conducted double screening at title and abstract, then at full text level to check for eligibility (e.g., compared perches versus control). The final list of studies underwent critical appraisal based on risk of bias and method validity, then data extraction. We used Hedges’ g as our effect size and fitted each outcome in a three-level meta-regression model. We also tested the effect of matrix type, bioregion, and precipitation variation as modifiers, and conducted sensitivity analysis based on risk of bias and method validity.

**Review findings:**

After screening, we accepted a total of 396 studies in 79 articles for the review. The majority of these studies examined seed (49%) and seedling density (28%) outcomes over richness, using mostly natural perches (68%) and, to a lesser frequency, artificial and semi-natural perches. Most studies that included distance to forest edge as a factor reported no effects (*n* = 68). We then analysed 333 studies in a meta-analysis. Results showed that natural perches had overall positive effects in increasing seed and seedling density and richness, while artificial and semi-natural perches were effective only for seed outcomes. We found high heterogeneity in our models, with perch effectiveness affected by matrix type, bioregion, precipitation variation, method specificity, as well as study quality. In general, perches showed robust positive effects in shrublands and grasslands in tropical, subtropical, and mediterranean biomes.

**Conclusions:**

The use of perches can be an effective ANR strategy to increase seed rain and seedling establishment in a variety of degraded landscapes. We recommend that natural perches be preserved in the matrix, but in areas lacking these natural features, to explore the use of artificial or semi-natural perches to increase seed rain and follow it up with additional treatments, such as soil amelioration and weeding, to improve seedling establishment. Due to insufficient data, we could not analyse the effect of distance to forest edge as a moderator in our meta-regression models. This gap can be addressed by examining perches placed at increasing distances from the edge and having better data sharing practices. We also emphasize a need for improving the quality of reporting, such as variances and detailed methodologies, in order for research to be useful for systematic reviews and meta-analysis.

**Supplementary Information:**

The online version contains supplementary material available at 10.1186/s13750-025-00363-8.

## Background

Deforestation is a major threat to biodiversity and important regulatory processes related to carbon, energy, and water cycles [1–3]. Globally within the last decade from 2010 to 2020, we experienced a net forest loss of 4.7 million ha per year [4] due to agricultural expansion [5], urbanization [6], and other anthropogenic pressures. There is a critical need and urgency to protect remaining forests and restore degraded areas, reflected in local and international commitments and declarations (e.g., New York Declaration on Forests, UN Decade on Ecosystem Restoration, COP26 climate summit). However, with the slow and little progress over the years, there are doubts that targets will be met [7, 8].

One potential hope to this challenge is the fact that forests can regenerate. Naturally regenerating forests (based on FAO categories) account for 93% of the world’s forest area according to the latest Forest Resources Assessment report [4]. Secondary forest regrowth often occurs after abandonment of agricultural fields and pastures, which is an increasing trend in Europe and North America due to socio-economic factors and farmland marginalization (i.e., declining profitability) [9, 10]. Given these, forest regeneration is touted as a low-cost solution to combat deforestation and mitigate climate change [11, 12]. Although secondary forests have been shown to exhibit rapid recovery of functioning, this natural succession process is dependent on intensity and time since past land use, as well as ecological factors [13, 14]. Some degraded areas can experience arrested succession due to high disturbance pressures from humans and herbivores, intense competition from grass / herbs / ferns / weeds, and low seed input, and thus would require interventions to allow forest regeneration [15, 16].

Reforestation activities can work with natural systems to accelerate natural forest recovery through Assisted Natural Regeneration (ANR) [11, 17]. One of the main principles of ANR strategies is to minimize disturbances (i.e., fire, livestock grazing) and competition (i.e., invasive species, weeds, vines) to the naturally regenerating trees [18–20]. ANR is an alternative to actively planting trees in degraded landscapes, which in many cases have limited success and proven ineffective in achieving ecological targets, thus failing to restore biodiversity and ecosystem functioning to levels observed in natural forests [21, 22]. Aside from minimizing disturbance and competition, ANR strategies can also focus on seed supply limitation by promoting seed dispersal by animals such as birds.

Seed dispersal in forests is often mediated by birds [23, 24]. Frugivorous birds, as well as omnivorous species, have been linked to forest regeneration, increasing the abundance and diversity of seed rain in degraded areas [25, 26], as well as plant species richness in forest gaps and regenerating forests [25, 27]. Seed passage through bird guts can also provide the added benefit of promoting seed germination and hence contributing to seedling establishment, although results have been varied and species-specific [28–30]. However, certain birds are hesitant to visit degraded areas due to greater risks of predation, harsher conditions, and lack of resources, hence several ANR strategies are aimed at attracting bird dispersers to the matrix [31]. These include the use of artificial perches [32], applied nucleation or tree islands [33], planting of fruit trees [34, 35], and other natural structures used by birds as perches, sometimes in conjunction with supplemental water [36] and playback luring [37].

The effectiveness of perches is mediated by the interplay of landscape and habitat factors with bird movement. There is clear evidence that birds move differently according to vegetation structure and spatial configuration of habitats in the landscape [38, 39]. We would, for example, expect that many birds are more likely to visit perches placed closer to the forest edge, perceived as less risky, than those farther away [40]. We also predict that birds are more likely to use perches located in more structurally complex areas compared to open (i.e., little to no trees), homogenous habitats (e.g., more birds using scrubland perches than grassland perches), because most frugivorous birds prefer to move in areas structurally akin to their native habitat (i.e., forest) [38]. Moreover, the matrix habitat can also interact with climatic conditions, particularly precipitation, to affect seedling establishment post-dispersal. The vegetation structure, at perches and around them, can alter water availability and soil moisture, known to affect the successful recruitment of seedlings from seeds [41].

ANR strategy using perches to attract bird dispersers requires consideration of the landscape and climatic factors, however the evidence base to inform perch effectiveness is scattered. To inform perch-related ANR strategies and facilitate the upscaling of ANR across regions, an evidence synthesis is needed to evaluate the effectiveness of various perching structures in degraded areas in promoting forest regeneration. These include natural elements that birds use as perches such as isolated trees and shrubs as well as artificial structures that humans have placed such as poles and fences. Guidetti et al. (2016) conducted a meta-analysis on the use of artificial perches in 2015, but they neither included natural perches nor examined moderator effects (e.g., matrix type, precipitation variation) [33]. On the other hand, Prevedello et al. (2017) did a global meta-analysis on the importance of scattered trees, a type of natural perch, but on a different set of outcomes, namely the species abundance and richness of selected taxa (i.e., plants, vertebrates, arthropods) [42]. This systematic review summarizes the importance of bird perching structures and discusses their effectiveness, ultimately to guide interventions using perches in future ANR programs.

### Stakeholder/expert engagement

We conducted an online stakeholder survey to seek external insights for our meta-analysis through emailing experts in the fields of forestry, ornithology, wildlife biology/ecology, restoration ecology, conservation science, and other relevant fields (Additional File 1). We received nine responses from participants who were affiliated with different universities (based in the UK, Thailand, and Philippines) and environmental conservation organizations (BirdLife International, Instituto Claravis, and Royal Society for the Protection of Birds). They provided suggestions for the inclusion of distance to forest as a modifier and for additional search terms, such as ‘forest*’ (locator), ‘scrub*’ (intervention), and ‘regenerat*’ (outcome).

### Objective of the review

This systematic review provides a comprehensive summary of the types of perches that can be used to attract bird dispersers for natural forest regeneration. We assessed the effectiveness of both artificial, natural, and semi-natural perches in promoting seed dispersal and seedling establishment by examining evidence from the literature and conducting a meta-analysis. We also tested if landscape and bioclimatic features are important covariates to observed effects. Seed germination as an outcome was not included, because it has been covered by Rogers et al. (2021) in their meta-analysis, which found that gut passage by birds increases seed germination [29].

#### Primary Question

How effective are natural, semi-natural, and artificial perches in promoting bird-mediated seed dispersal and seedling establishment in degraded landscapes? (Table 1)

#### Secondary question

How do landscape and bioclimatic features alter the effectiveness of natural, semi-natural, and artificial perches?

**Table 1:** The PICO structure of the systematic review research question

Question key elements	
Population (P)	Degraded areas near a forest
Intervention (I)	Artificial perches (e.g., wooden posts, wires) Natural perches (e.g., single trees, shrubs, rocks) Semi-natural perches (e.g., perches artificially constructed using dead branches, exhibiting natural plant architecture)
Comparator (C)	Temporal: before and after intervention at the same site Spatial: with and without intervention at adjacent sites with the same expected seed source and ecoregion, comparing them within the same time period
Outcome (O)	Seed richness Seed density Seedling richness Seedling density
Moderator	Matrix type (e.g., open cleared area, grassland, shrubland) Distance of perch to forest edge Precipitation variation (very dry, dry, normal, wet, very wet) Bioregion / biome Risk of Bias score based on CEE Critical Appraisal Tool Version 0.3 [43] (low, medium, high) Methods validity score (low, high)

## Methods

This review follows the published protocol with some deviations [44], as well as the RepOrting standards for Systematic Review Syntheses (ROSES) [45], and the PRISMA -EcoEvo checklist [46] (Additional File 6 and 7).

### Deviations from protocol

We included a full Boolean-style search string ‘(bird) AND (perch* OR tree OR shrub OR wire* OR post*) AND (seed dispersal OR seed rain OR seedling* OR regenerat*) AND (forest* OR woodl*)’ for the Google Scholar search, in addition to the simpler string ‘Bird AND perch AND seed dispersal’ indicated in the protocol, to increase the comprehensiveness of our search (Additional File 2). We decided to relax the eligibility criteria pertaining to the population and locator aspect of the question. First, we included articles that alluded to bird dispersers; some studies did not explicitly mention avian dispersers but considered, in general, plant species that had zoochorous mode of seed dispersal. We took into account the specificity of the observed seed/seedling in the method validity scoring for subgroup analysis, ranking studies with certain bird-dispersed data as high and those with general dispersal mode as low validity. Secondly, we included studies that were conducted in or near shrublands, instead of restricting to areas near forests. Although our question was formulated with forest regeneration in mind, studies that looked at seed dispersal in or near shrublands can still provide evidence on how perches can increase seed and/or seedling inputs to degraded landscapes. In addition, one study [47] examined effect of isolated trees on seed dispersal in the context of woodland encroachment to a savanna, which we decided to include as the data provided useful comparison of seedling establishment under perch (i.e., isolated tree) versus away from perch (i.e., open area near tree). During the screening process, instead of discussing the conflict with the whole team (four reviewers) as planned in our protocol, the two reviewers first discussed the articles with conflicting decisions to check for misunderstanding and only when no resolution was reached that we consulted the whole team.

At the data extraction step, we refined the predefined options in the datasheet to make it more appropriate for the type of studies considered. Under intervention type, instead of just having ‘artificial perch’, we specified whether the perch was either ‘artificial pole’ for classic standing structures or ‘artificial other perch’ for wires and fences. In addition, we have added a ‘semi-natural’ category for perches artificially constructed using dead branches. At the critical appraisal step, we also revised the method validity assessment to having scores of low, medium, and high to correspond to the certainty and specificity of the measured outputs being bird dispersed (see section on study validity assessment).

We have modified the quantile thresholds to classify the moderator ‘precipitation variation’, on whether the study was conducted under very wet, wet, normal, dry, or very dry. In the protocol, we initially planned to use the closest weather station for each study site, but due to the sparse coverage of monitoring stations in many regions as well as the difficulty of accessing data from each of them, we decided to use the CHIRPS dataset, which has standardized rainfall estimates that have been validated in many countries [48–50] 

For the meta-analysis itself, we found high variances in our effect sizes and thus decided to include another sensitivity analysis to explore the effect of outliers in the meta-analysis (see methods). We were not able to compute other measures of heterogeneity aside from I^2^ (i.e., DerSimonian-Laird, Paule-Mandel, and Sidik-Jonkman) as they were not applicable to multilevel models. Lastly, we opted not to present the ‘corrected’ estimates resulting from the publication bias test [51], as the high heterogeneity in our models and inaccuracy of correction (i.e., can be under and overestimated) make these ‘corrected’ estimates unreliable and difficult to interpret.

### Search strategy and search strings

We searched for articles in eight predefined databases (Table 2) and supplemented them through solicited calls for relevant papers and references in the published review by Guidetti et al. (2016) [33]. The searches were conducted from 7th to 8th of September 2023, whilst the public call for relevant papers was posted on the 13th of September 2023.

**Table 2:** The final list of search sources

Source	Institutional subscription	Search fields
**Bibliographic sources**		
Web of Science Core Collection • Science Citation Index Expanded (SCI-EXPANDED) 1970-present • Social Sciences Citation Index (SSCI) 1970-present • Arts & Humanities Citation Index (AHCI) 1975-present • Conference Proceedings Citation Index– Science (CPCI-S) 1990-present • Conference Proceedings Citation Index– Social Science & Humanities (CPCI-SSH) 1990-present • Emerging Sources Citation Index (ES—) -2015-present	Newcastle University	Topic (includes title, abstract, author keywords, and keywords plus)
Zoological Record	Newcastle University	Topic (includes title, book title, abstract, broad terms, descriptors data, super taxa, systematics, taxa notes)
SciELO Citation Index	Newcastle University	Topic (includes title, abstract, author keywords)
Scopus	Newcastle University	Article Title, Abstract, Keywords
CAB Abstracts	Newcastle University	WOK Free-Text index (English Item Title, Original Item Title, Source Abstract, CABICODE Names, Descriptors, Organism Descriptors, Geographic Location, Identifiers, Broad Terms)
ProQuest Natural Science Collection	Newcastle University	Title, Abstract, Keywords
**Website**		
Conservation Evidence	Open Access	NA Search studies using keyword “perch” and under category “birds”
**Web-based Search Engine**		
Google Scholar	Open Access	NA (bird) AND (perch* OR tree OR shrub OR wire* OR post*) AND (seed dispersal OR seed rain OR seedling* OR regenerat*) AND (forest* OR woodl*) Bird AND perch AND “seed dispersal”

Briefly, the final search string included terms for birds as population, natural and artificial perches as interventions, seed dispersal and seedling establishment measurements as outcomes, and lastly forest or woodland as locators, which were combined with an “AND” operator to form the final string: (bird* OR avian OR aves OR disperse*) AND (palm* OR fruit* OR perch* OR “artificial perch*” OR roost* OR nucleation* OR nuclei OR “tree isl*” OR “woodland isl*” OR “habitat isl*” OR “remnant tree*” OR “isolated tree*” OR “single tree*” OR shrub* OR wire* OR post* OR scrub*) AND (“seed dispers*” OR “seed rain*” OR seedling* OR regenerat*) AND (forest* OR woodl*). The search fields included title, abstract, and keywords for all databases, except for the two web-based databases, Conservation Evidence and Google Scholar, which operate their own search algorithms. We restricted our search to publications in English, including literature with abstracts published in English but using other languages in the full text. We acknowledge that our search strategy could have missed regional studies, but we tried to limit this bias by including references cited in review papers.

We then removed the duplicates across search results from different databases based on DOI and title matches using the R package ‘revtools’ [52] and further refined it using the de-duplication algorithm in the software Rayyan [53]. We kept the amassed library at Rayyan for the screening steps.

### Article screening process and eligibility criteria

The screening was done at two levels– first at title and abstract, then at full-text stage– using the pre-defined eligibility criteria as in protocol, with some minor deviations as stated above. We (JG, MW, SD, SD) double-screened all studies at both stages and reconciled conflicts as the screening progressed following best practice guidelines [54]. The disagreement rate was computed as the proportion of studies with conflicting decisions between two independent reviews. Disagreement was low (max: 4.19% of 716 studies) for title and abstract filtering stage, but it was high (max: 24.2% of 33 studies) for the initial full-text screening and thus triggered refinement of the criteria (see above section on Deviations), with later screenings having lower disagreement rates (< 10%). Conflicts were first reviewed by the two reviewers involved, and thereafter through discussion with the whole team in case of unresolved conflicts. None of the review team members had articles included in the review. We used the following criteria to assess inclusion of studies in our review:

#### Subject

Articles that examined seed dispersal and seedling establishment by bird dispersers, including articles that alluded to dispersal by birds.

#### Intervention

Articles that identified and collected data from natural, semi-natural, and artificial perching sites.

#### Comparator

Articles compared sites with perch with adjacent control sites but without perch. The with perch and without perch sites must have similar conditions and have replicates.

#### Outcome

Articles reported raw data, descriptive and/or inferential statistics in Figure, Table, or Text on one of the following: seed richness, seed density, seedling richness, and seedling density.

#### Locator

Studies that were conducted in degraded areas near forest, but also in other habitats such as shrublands and coastal dunes.

#### Language

Articles written in English.

A key component for eligibility is the comparison that the studies made, which requires a control versus intervention type of study design. As an example of a valid study, in a landscape with open areas beside a forest, researchers measured seed rain under 10 isolated trees in the open area and compared that with seed rain collected in 10 plots under open sky a few metres away from the trees (control). Both intervention and control samples are in the same landscape, experiencing similar biotic and abiotic conditions and sharing the same seed source from the nearby forest. Using this eligibility criteria ensures the alignment of the studies to our review questions.

### Study validity assessment

We assessed the quality of each study that passed the screening stage based on risk of bias and method validity. We used the CEE Critical Appraisal Tool Version 0.3 [43] which had seven sub-criteria to determine whether the overall risk of bias was low, medium, or high. We used Criteria 3 for observational studies involving pre-existing natural perches (e.g., isolated trees, shrubs) and Criteria 4 for experimental studies that modified or added perches (e.g., artificial posts, semi-natural poles). Overall risk of bias was considered high if the study scored high in at least one criterion, medium if it scored at least one medium but no high, and low if it scored low for all criteria. In addition, we also assessed method validity to classify studies based on whether their outcomes are bird-specific or not (Table 3). Studies with high validity employed methods that only considered bird-dispersed seed or seedlings, those with low validity obtained outcomes that are generic (i.e., various modes of dispersal), and those with some support as medium.

**Table 3:** Study quality assessment based on the external validity of methods

Validity Score	Criteria
**Methods to measure species richness and density of bird-dispersed seeds**	
high	Field observation of seed dispersal by birds on perches; Seed rain collection representing dispersal by birds (i.e., daytime only to exclude bats, excluded non-bird dispersed species through method); classification of bird-only dispersed species
medium	Some evidence that the output is mainly bird-dispersed (i.e., observations, use of faeces, post-classification of the seed species as bird-dispersed)
low	Seed rain collection representing dispersal by animals and non-specific to birds (i.e., open at night, non-exclusion of other potential dispersers)
**Methods to measure seedling species richness and density**	
high	Long-term monitoring of plots under perches with known seed rain data and with predator and herbivore exclusion set-up; classification of bird-only dispersed species
medium	some evidence presented to support that it is mainly bird-dispersed
low	Seedlings dispersed by animals in general and non-specific to birds (i.e., sampling under perch sites without known seed rain data)

We used both overall risk of bias and method validity scores as subgrouping variables in the meta-analysis to check the sensitivity of the results and to frame our results and conclusion with consideration of the risk of bias and limitations. All assessments were cross-checked by a second reviewer and resolved between the two, or by the entire team if no resolution was reached.

Lastly, to assess external validity, we extracted the study context, such as where the study was conducted and what kind of perch they used. We summarise the representation captured in the narrative synthesis to provide an insight into the review’s generalisability. In addition, we tested the effect of biome and perch type in the meta-analysis to determine whether results are robust across different settings.

### Data coding and extraction strategy

We initially started on data extraction using the pre-designed datasheet published in the protocol, however we had to modify some of the coding options to accommodate the information encountered in the accepted studies (see Additional File 3). For studies that did not provide the geographical coordinates of their study sites, we used Google Maps to obtain the longitude and latitude of the most specific place name provided in their descriptions (noted under ‘coord_source’ in the dataset). The accuracy of the estimated coordinates is deemed not of critical importance, as they were used primarily for collecting remote sensing data that are of relatively low resolution (~ 5 km), allowing room for errors.

Outcome-related data (i.e., seed/seedling richness/density) were taken from the text, figures, and tables in the publication. We used the metaDigitise R package [55] to extract values from figures such as bar plots and box plots, and an Excel spreadsheet calculator to estimate mean and variance from sample size, median, range and/or interquartile range [56]. If the data presented was incomplete (i.e., no variance) or unclear, we emailed the corresponding author/s to ask for the missing data or for clarification. Some studies classified and reported seed rain data based on seed dispersal mode, type of plant (e.g., shrub, tree), and species residency status (e.g., native or non-native). For such cases, we only used those specific to animal or bird dispersal, to seeds of forest tree species, and to native species, respectively, because these were the relevant outcomes to our question. As the number of accepted articles was large (i.e., exceeded 50), the data from each article was extracted by one primary reviewer and cross-checked by a secondary reviewer.

### Potential effect modifiers/reasons for heterogeneity

We considered matrix type, distance of perch to seed/seedling source, precipitation variation, and bioregion as our effect modifiers. The first two variables provide important landscape level context because of their role in facilitating or hampering movement of birds through the landscape [38, 39]. In general, forest birds tend to stay within or near vegetated areas and are reluctant to venture into matrix habitats (i.e., semi-open or open areas) due to increased predation risk and reduced resource availability [38]. Therefore, we expect that the probability of forest birds to cross the matrix is inversely associated with gap width (defined as distance from one patch to the nearest patch), and that the likelihood of birds to use perches is higher closer to forests overall. Matrix type can affect movement decisions of birds by influencing the cost and benefit of movement steps when travelling between patches [57]. Matrix habitat comprising dense, tall vegetation for example, can provide cover from predators (lower cost) but can also limit visual perception of the landscape (higher cost). Whilst matrix habitat of similar structure to habitat associated with a specific bird species has been confirmed to allow for increased movement rates in a recent meta-analysis [38].

We considered rainfall variation as potential modifiers for the effect size estimates on seedling establishment outcomes because of the role of water availability in seed germination and survival [41]. We downloaded annual precipitation data from the Climate Hazards Group Infrared Precipitation with Stations dataset (CHIRPS; Funk et al., 2015). CHIRPS provides gridded rainfall data derived from both rain gauge stations and satellite data, covering daily data from 1981 to near present with spatial resolution of approximately 5 km (0.05°). We obtained the total annual rainfall in millimetres from 1981 to 2023 for each site to calculate its historical rainfall distribution. We then identified whether the year when the study was performed fell within very dry (< 15th percentile of the historical rainfall), dry (15th– 35th ), normal (35th– 65th ), wet (65th– 85th ) and very wet year (> 85th percentile) [59] (Additional File 3). The few studies which did not state year of study (*n* = 27; 3 articles) were excluded from the analysis.

We also examined whether effects varied among bioclimatic regions, which differ due to differences in geology, biodiversity, disturbance regimes, and land use history. We used the Ecoregions2017©^Resolve^ map [60] to classify study sites into biomes (i.e., Mediterranean Forests, Woodlands & Scrub, Montane Grasslands & Shrublands, Temperate Broadleaf & Mixed Forests, Temperate Conifer Forests, Temperate Grasslands, Savannas & Shrublands, Tropical & Subtropical Grasslands, Savannas & Shrublands, Tropical & Subtropical Dry Broadleaf Forests, Tropical & Subtropical Moist Broadleaf Forests). Lastly, we evaluated to what extent the quality assessment scores (method validity and CEE Critical Appraisal Tool) influenced estimates of effect size.

### Data synthesis and presentation

#### Descriptive statistics and narrative synthesis

All articles after critical appraisal stage were included in the narrative synthesis. All information presented, including the meta-data of each study, are available in the Additional File 3.

#### Quantitative synthesis

Given that enough data was obtained, we conducted a meta-analysis using the subset of studies with complete data. Only articles that reported the means and variances for both control and intervention groups were included in the meta-analysis. We computed the unbiased standardized mean difference Hedges’ *g* [61] as our measure of effect sizes:

$$\:g=\:\frac{{X}^{E}-\:{X}^{C}}{s}\:J$$

representing the difference in the mean (*X)* of the experimental (E) and control group (C), standardized by the pooled standard deviation (s) and includes a correction factor (*J*) for small sample size (49) which are computed as

$$\:s\:=\:\sqrt{\frac{\left({n}_{E}-1\right)\:{s}_{E}^{2}+\:\left({n}_{C}-1\right)\:{s}_{C}^{2}\:}{{n}_{E}+\:{n}_{C}-2}}$$

$$\:J=1-\:\frac{3}{4\:\left({n}_{E}+\:{n}_{C}-2\right)-1}$$.

where $\:{s}_{E}$ and $\:{s}_{C}$ correspond to standard deviations, and $\:{n}_{E}$ and $\:{n}_{C}$ to sample sizes of the two groups. The effect size variance ($\:{v}_{d})\:$will be obtained, using

$$\:{v}_{d}=\:{J}^{2}\:\:\left(\frac{{n}_{E}+\:{n}_{C}}{{n}_{E}{n}_{C}}+\:\frac{{g}^{2}}{2\:\left({n}_{E}+\:{n}_{C}\right)}\right)$$.

We estimated the effect sizes for two seed-related outcomes (i.e., seed density and seed richness) and two seedling-related outcomes (i.e., seedling density and seedling richness). Positive *g* values indicate higher seed richness, seed density, seedling richness, and seedling density in areas with perches than in those without perches, and vice versa.

To handle effect size dependency, we used a multilevel random-effects meta-analytical model approach with study identification as a random effect. We reported the heterogeneity as I^2^ following Nakagawa & Santos (2012) [62], which is the proportion of variance between effect sizes that can be attributed to real heterogeneity and not due to sampling error. We then examined the distribution of the total variance across the levels and compared the three-level model with null models, where the variance of one or both levels is held constant (i.e., within-study variance constrained, between-study variance constrained, and both level variance constrained), following the approach described in Assink and Wibbelink (2016) [118]. We used ANOVA and AIC values to evaluate the fit of the four models.

We assessed the effect of potential modifiers through a mixed-effects meta-regression approach. We fitted the effect size estimates for seed-related outcomes to matrix type and bioregion as fixed effects, and similarly for the seedling-related outcome but with the addition of precipitation variation in uni-moderator models. All computations were done in R Statistical Software version 4.4 [63] using the metafor package [64], while visualization with orchard plots was done using the orchaRd package [65]. Orchard plots were favoured over forest plots because the former displays not only overall estimates (i.e., meta-analytic means) and their confidence intervals, but also prediction intervals and individual effect sizes to better present heterogeneity.

Publication bias, when certain subsets of studies (e.g., statistically significant results) are more likely to be published, was tested using multilevel meta-regression following Nakagawa et al. (2022) [51]. We selected this test given its performance in handling heterogenous and non-independent data, fitting meta-regression models with the square root of the inverse of effective sample size and publication year as moderators to primarily detect small-study effects and time-lag bias [51]. Small study effects, the discordance between effect sizes of studies with smaller sample sizes compared to larger ones, can arise due to publication bias, but this bias can also occur due to random variability and selective outcome reporting [66]. In addition, we also performed a sensitivity analysis by removing outliers or influential studies from the meta-analysis. Outliers were identified as such if the study’s confidence interval overlapped with the confidence interval of the pooled effect [67].

## Review Findings

**Fig. 1:**
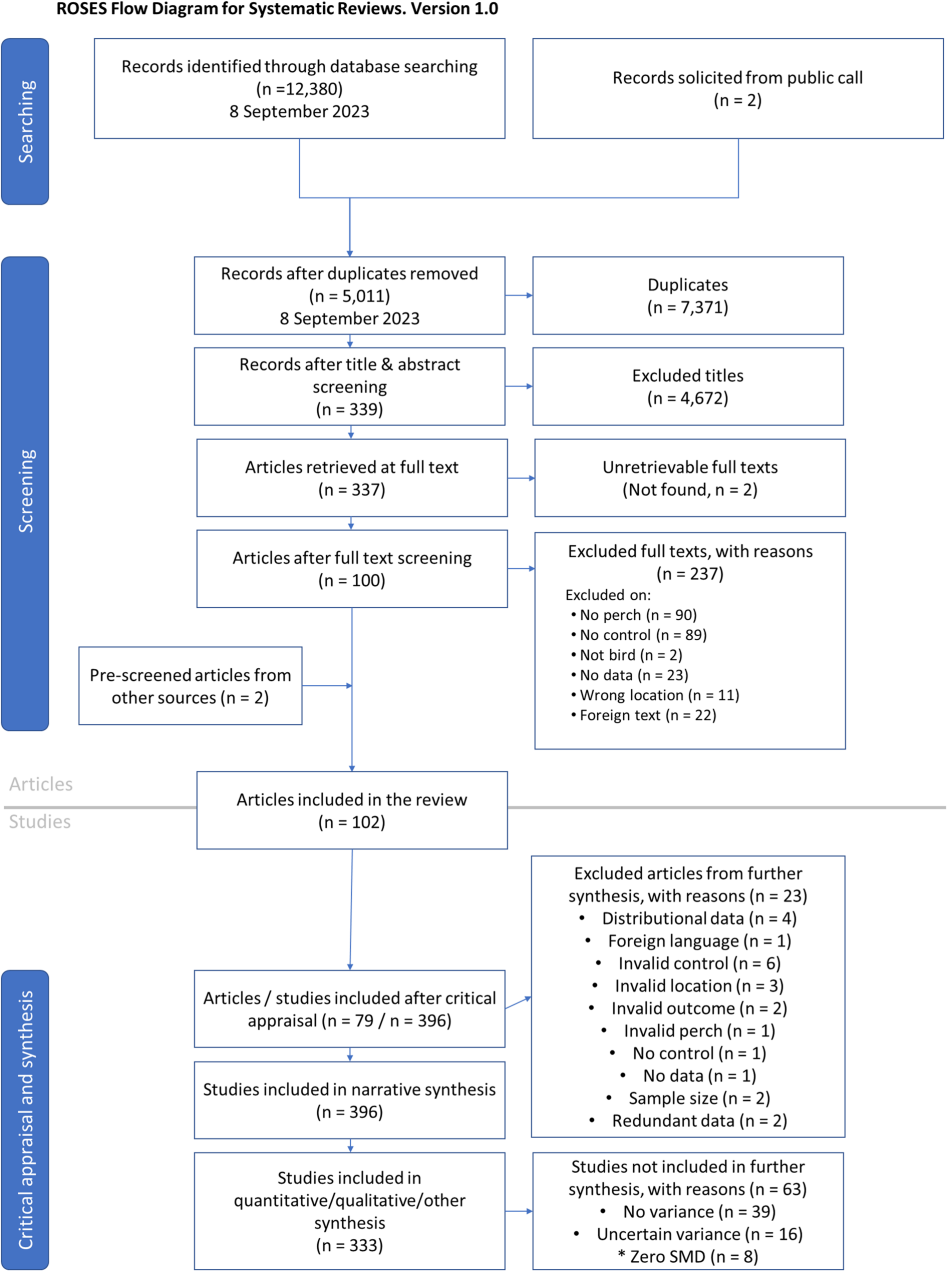
The ROSES Flow Diagram for the systematic review showing the number of articles and studies accepted through each stage. Studies refer to separate datasets extracted from accepted articles

From an initial search result of 12,380 articles collated from the bibliographic databases, we found 102 articles to include in the review after removing duplicates and conducting the screening (Fig. 1). However, 23 articles had to be removed after critical appraisal because of redundant data and issues concerning study design and data validity (see Additional File 2 for reasons for exclusion). A total of 396 studies were included in the narrative synthesis, of which 333 studies were eligible for quantitative analysis (Additional File 3).

### Narrative synthesis

**Fig. 2:**
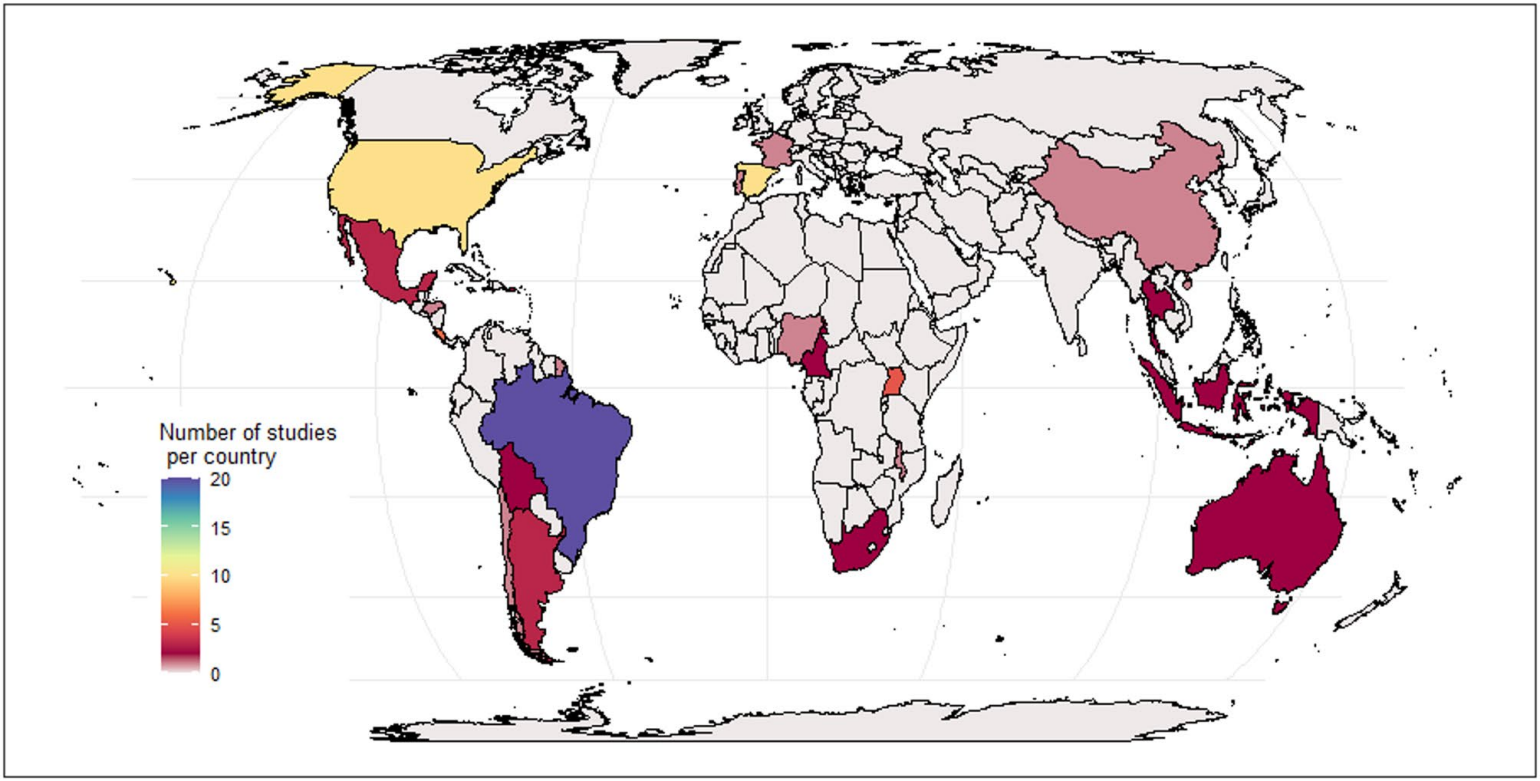
Global map of the location of studies included in the review. The colour gradient represents the number of studies conducted in the country. Majority of the studies came from the Neotropics, with Brazil contributing the highest number of studies, while most of Afrotropic and Palearctic region are understudied

A total of 396 studies from 79 articles were included in the narrative synthesis. Studies were mainly published in journal articles (97.7%), with a few theses (1.52%), book Chaps. (0.51%), and one conference proceeding (0.25%). Most were from the Neotropics (201 studies, 38 articles), followed by Palearctic (59 studies, 12 articles), Afrotropic (57 studies, 11 articles) and Nearctic (38 studies, 8 articles) (Fig. 2). Very few studies were represented in the Australasia (11 studies, 2 articles), Oceania (16 studies, 3 articles), Indomalayan region (14 studies, 5 articles). Despite representation across realms, studies were spatially concentrated in 22 countries, with a disproportionately large proportion conducted in Brazil (25.3%), followed by Spain (12.7%) and the United States of America (12.7%). The Afrotropic and Palearctic regions have only been studied in selected nations, leaving most of the region unrepresented.

**Fig. 3:**
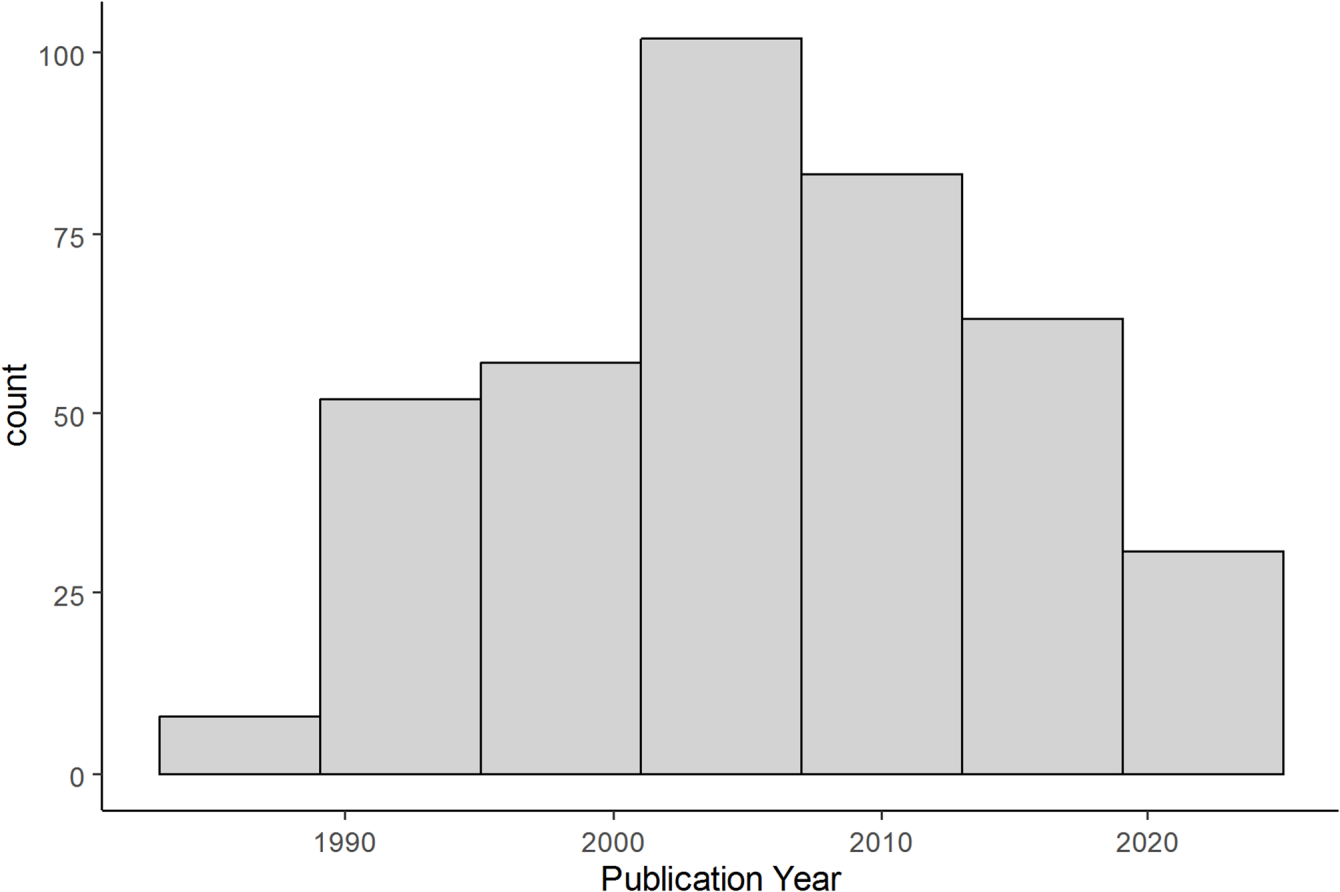
The distribution of year of publication represented in the review. Scientific interest on the effect of perches on seed dispersal and seedling establishment peaked in the 2000s

In terms of biome, most studies were conducted in forests, namely Tropical & Subtropical Moist Broadleaf Forests (216 studies, 44 articles), Mediterranean Forests, Woodlands & Scrub (82 studies, 13 articles), Temperate Broadleaf & Mixed Forests (30 studies, 7 articles) but few in Temperate Conifer Forests (6 studies, 1 article) and in Tropical & Subtropical Dry Broadleaf Forests (10 studies, 3 articles), while scrublands and grasslands were less represented (Montane Grasslands & Shrublands: 8 studies, 1 article; Temperate Grasslands, Savannas & Shrublands: 26 studies, 5 articles; Tropical & Subtropical Grasslands, Savannas & Shrublands: 18 studies, 5 articles). The oldest publication was in 1983, while the newest was in 2022 (Fig. 3). No trend was evident in terms of the number of studies being published across years.

**Fig. 4:**
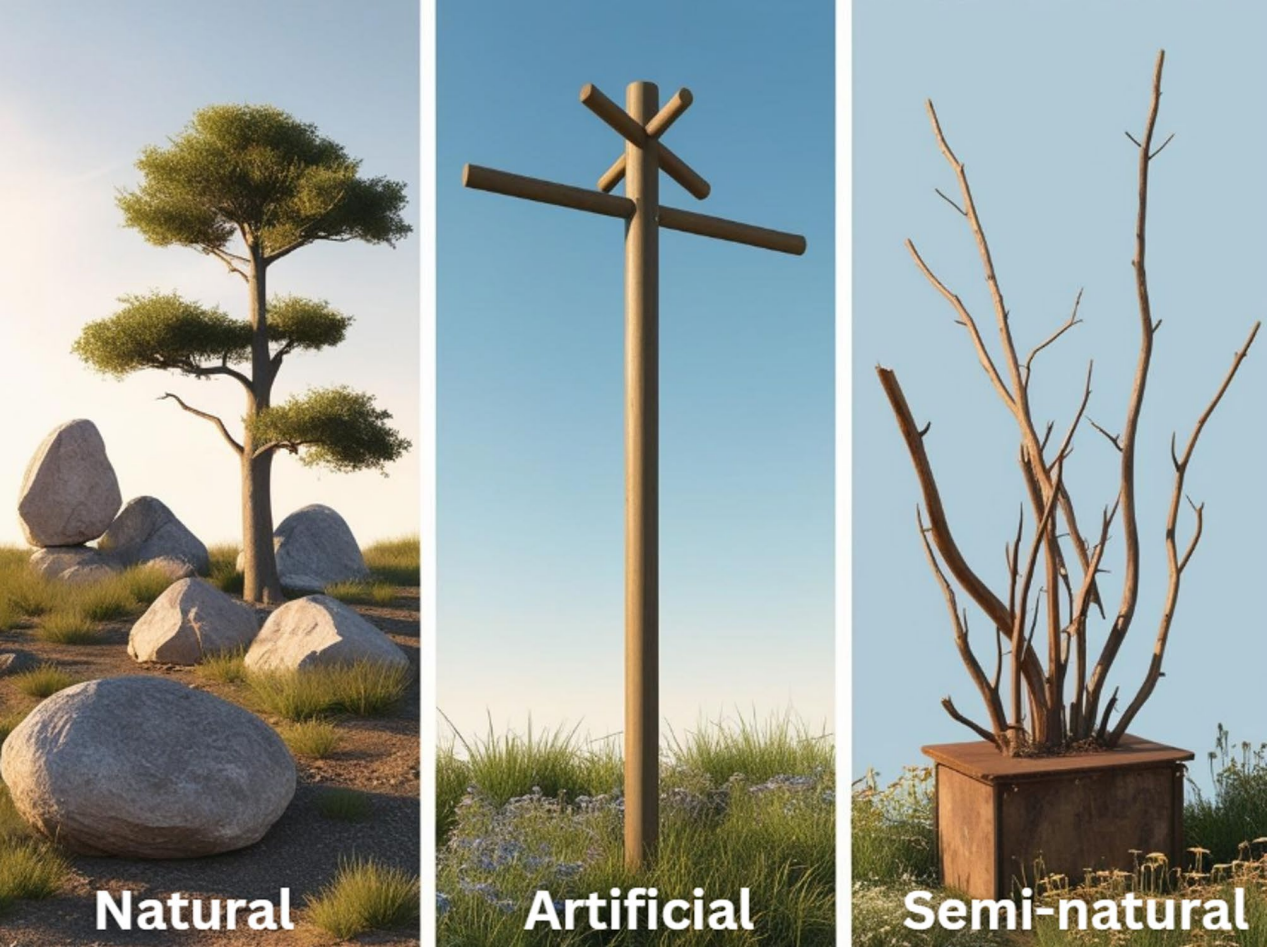
Illustrations of the three different perch types examined in the studies. Illustrations were made with the use of artificial intelligence on Canva (Magic Studio™), guided by author description

We noted different types of artificial, semi-natural, and natural perches (Fig. 4). Artificial perches, tested in 90 studies (22.7%), were mainly straight poles made from wood (e.g., bamboo) or in one study made from PVC pipes with horizontal bars and crossbars. We also found a few studies that examined live fences (i.e., living trees tied with wire), brushwood, and wood piles, which were also considered artificial. Meanwhile, natural perches were examined in 270 studies (68.2%), and we found a variety of types from shrubs to trees to tree islands, as well as two studies considering rock/stone. We have also noted semi-natural perches in 36 studies (9.1%), those made from dead branches/snags propped up with support.

There were more studies on seed rain than on seedling establishment, and more on density than on richness outcomes. Out of the 396 studies, almost half (49.5%) reported seed density between control and perch set-ups, 28.0% on seedling density, 13.6% on seed richness and only 8.84% on seedling richness.

Distance to forest edge was one of the most examined covariates, but even then, only 19.9% (79 studies) of the total tested for its effect on seed rain and/or seedling establishment. Majority (68 studies) of them found no effect from distance to forest edge. Only four studies, described in two articles, mentioned positive relationships. Castilhos De Freitas et al. (2022) placed artificial perches 5 m, 25 m, and 50 m away, and they found seed richness and density both increased with greater distance from the forest edge. Another study by Slocum & Horvitz (2000) [69] examined different tree species as natural perches and got mixed results; the positive relationship was true for one species, *Cordia alliodora*, but other tree species had either negative or insignificant correlations between seed density and richness with distance across different tree species. In contrast, six articles reported an inverse relationship. Dimitri & Longland (2017) [70] found less Western Juniper (*Juniperus occidentalis*) seeds under shrubs and tree microsites located at farther distances from the woodland edge. Similarly, a negative relationship with seed density was reported by Sritongchuay et al. (2014) [71] in Southern Thailand and Jaafar et al. (2021) [117] in Nigeria examining trees as perches, Gale et al. (n.d.) [72] in Northern Thailand studying artificial perches at distances ranging from 24 m to 480 m, and Parejo et al. (2014) [40] in Portugal investigating semi-natural poles placed at 100 and 300 m distances. For seedling-related outcomes, Siqueira et al. (2017) [74] found decreasing sapling density with increasing distance from forest edge.

Out of the 396 studies, 59.3% were assessed to have a high risk of bias, while the rest were assessed as having a medium risk of bias. No studies scored as having low risk. For method validity, the 250 studies that had seed-related outcomes were spread across low (36.4%), medium (34.4%), and high (29.2%) validity scores, while the 146 seedling studies had low (45.9%), medium (20.5%), and high (33.6%) scores.

### Meta-analysis

We gathered sufficient data to analyse the effect of perches on all four outcome types. After excluding studies that did not report variance at all (*n* = 39), did not clarify what variance was presented (*n* = 16), and zero data studies (*n* = 8), we had a total of 333 studies that were useful for the meta-analysis. Of which, 160 studies reported seed density, 53 for seed richness, 89 for seedling density, and 31 for seedling richness.

We used three-level models for all four outcomes after conducting comparison tests (Table 4; see Additional File 4 Table 1 for likelihood ratio test results and AIC scores), with study identifier as level 2 and article identifier as level 3 variance. There was a significant positive effect estimate on seed density and richness across all three types of perches, but only natural perches were effective in increasing the seedling density and richness (Table 4). We observed high levels of heterogeneity in the effect sizes within our data as shown by the high I^2^ (range: 84–98%) for all four models.

**Table 4:** Pooled effect sizes (Hedges’ g standardized mean difference) with lower and upper 95% confidence intervals (CI) and prediction interval (PI) showed positive effects of different types of perches on four seed and seedling outcomes based on three-level meta-analysis model with random effects (article and study ID). K indicates sample size and I^2^_total is the percentage of variance explained by heterogeneity. Significant results (*p* < 0.05, confidence intervals not crossing zero) indicated by asterisk (*) beside estimate

Outcome	Perch Type	k	g	CI	PI	I^2^_total
seed density	Artificial	28	1.21 *	[0.77;1.65]	[-0.50; 2.92]	91.00%
	Natural	116	1.22 *	[0.94;1.51]	[-0.46;2.90]	
	Semi-natural	16	1.59 *	[0.98;2.21]	[-0.17;3.36]	
seed richness	Artificial	12	1.97 *	[0.51;3.43]	[-3.03;6.96]	98.00%
	Natural	36	1.68 *	[0.38;2.97]	[-3.27;6.63]	
	Semi-natural	5	8.60 *	[5.75;11.44]	[3.04;14.15]	
seedling density	Artificial	13	0.80	[-0.02;1.62]	[-1.33;2.94]	91.51%
	Natural	66	1.22 *	[0.79;1.64]	[-0.80;3.23]	
	Semi-natural	10	0.81	[-0.05;1.68]	[-1.34;2.96]	
seedling richness	Artificial	7	0.52	[-0.52;1.55]	[-1.38;2.41]	84.65%
	Natural	24	0.89 *	[0.36;1.41]	[-0.79;2.56]	

#### Seed density

Perches had a significant effect of increasing seed density for all three perch types (Table 4). We found a high heterogeneity of I^2^ = 91.00% and between-study variance σ = 0.63. The study level and article level explained 11.20% and 79.81% of the total heterogeneity, respectively. In the three-level model without modifiers, the general effect of perches on seed density was similarly positive (k = 160, g = 1.26, 95% CI = 1.02–1.50) (Additional File 4 Table 2).

We conducted a sensitivity analysis using the risk of bias and method validity as moderators. Seed density studies in our meta-analysis fell under high (55.62%) and medium (44.37%) risk of bias (see Additional File 4 Fig. 1 for the breakdown of each criterion), and we found consistent positive effect of general perches (i.e., all types considered) in both categories (range of g: 1.18–1.30) (Additional File 4 Table 3). However, effects were inconsistent for perch types across different risk of bias subgroups; only natural perches showed consistent positive effects, while the other two types resulted to significant effects only with the high-risk studies (Additional File 4 Table 4). For method validity, we found 60 studies (37.50%) with high, 58 (36.25%) with medium, and 42 (26.25%) with low validity, and the effect sizes for perches in general also remained significant and positive across these three levels (range of g: 1.20–1.68) (Additional File 4 Table 5). However, when considering different types of perches, semi-natural perches had mixed effects i.e., significant with medium subgroup but nonsignificant with high and low subgroup (Additional File 4 Table 6).

**Fig. 5:**
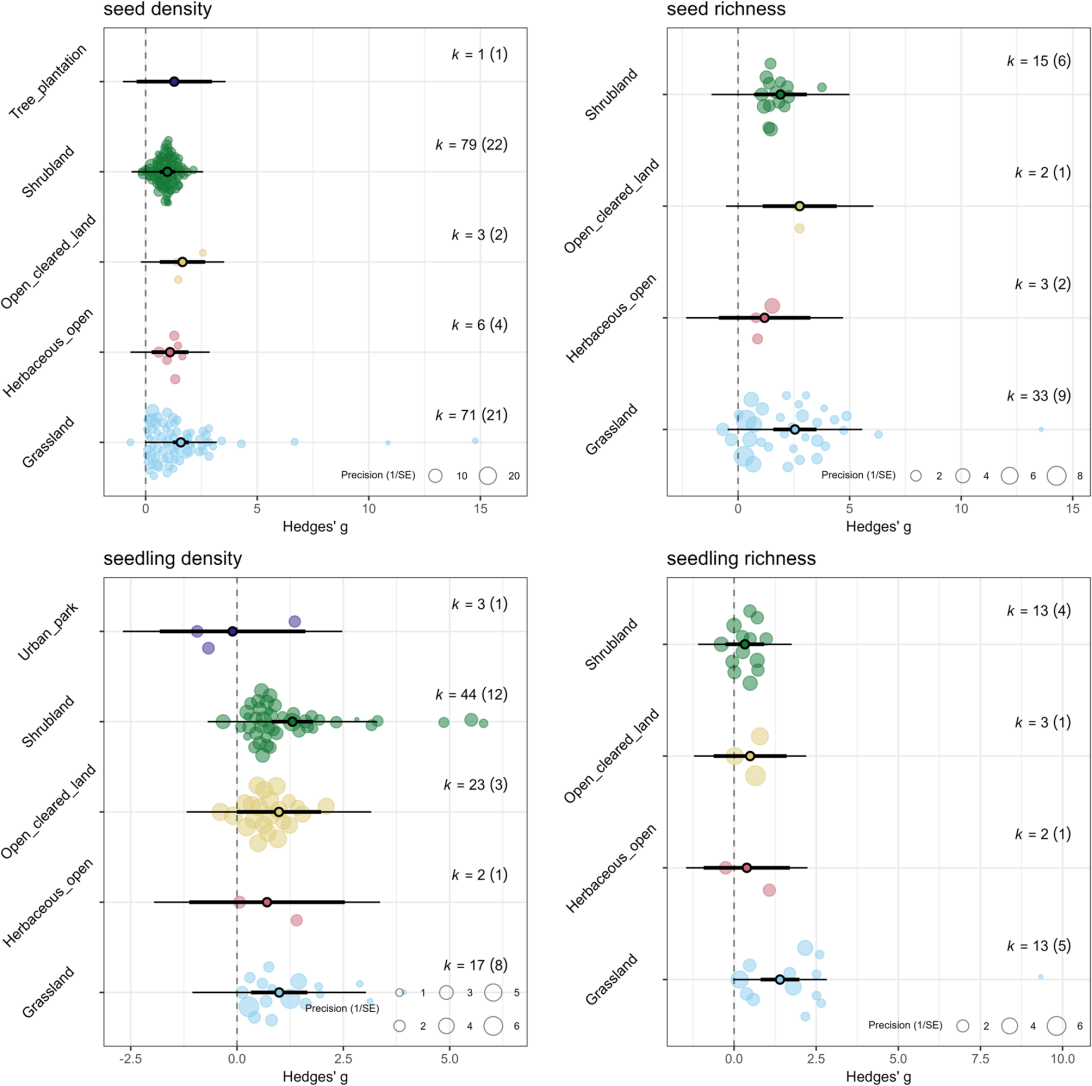
Orchard plot for the standardized mean difference (Hedges’ g) effect size on seed density (top left) and richness (top right), and seedling density (bottom left) and richness (bottom right) across different matrix types. Individual effect sizes (circles) of each study are scaled by their precision (the inverse of standard error). k is the number of effect estimates or the number of studies considered, with the number of articles indicated within parentheses. See model results summary statistics in Additional File 4 Table 7

The matrix type where perches were placed influenced perch effectiveness (Fig. 5). We examined studies that had perches in grasslands (k = 71), herbaceous open areas (*n* = 6), open cleared land (k = 3), shrubland (k = 79), and tree plantation (k = 1). Perches were effective across all matrix types, with greatest effect in cleared lands (g = 1.65, 95% CI = 0.64–2.67) and grasslands (g = 1.58, 95% CI = 1.22–1.93), followed by herbaceous open (g = 1.09, 95% CI = 0.27–1.92) and least in shrubland (g = 0.98, 95% CI = 0.64–1.32), but note that cleared lands and herbaceous open have insufficient sample sizes from which we can draw conclusions (Additional File 4 Table 7). Heterogeneity I^2^ of the model was high at 90.14%, with between-study variance σ of 0.55. Subgroup analysis according to risk of bias showed consistent positive effects for grasslands and shrublands (Additional File 4 Table 8).

To determine if the effect we found was affected by bioregions, we tested biome as a modifier in the model and found significant positive perch effects on seed density for six of them, only showing nonsignificant effects in Temperate Conifer Forest (k = 6, g = 0.65, 95% CI= -0.89-2.20) (Table 5). Perches were effective in Mediterranean Forests, Woodlands & Scrub, Temperate Broadleaf & Mixed Forests, Temperate Grasslands, Savannas & Shrublands, Tropical & Subtropical Dry Broadleaf Forests, Tropical & Subtropical Moist Broadleaf Forests, and also Tropical & Subtropical Grasslands, Savannas & Shrublands (but note very small sample size, *k* = 4, and hence inconclusive). There was high heterogeneity in the models with I^2^ = 90.61%, contributed by article level (78.22%) and study level (12.39%) heterogeneity, and with between-study variance σ = 0.59. However, upon conducting a sensitivity analysis using risk of bias scores, only Tropical & Subtropical Moist Broadleaf Forests showed consistent positive effects, while the effect of perches was only significant in Temperate Broadleaf & Mixed Forests and Temperate Grasslands, Savannas & Shrublands for high-risk studies but not for medium-risk subset. Other biomes could not be compared as sample sizes were too low (Additional File 4 Table 9).

**Table 5:** Results of the three-level models for the four seed and seedling outcomes with biome as moderator. Biome classification was based on the Ecoregions2017©^Resolve^ map [60]. Effect estimates are presented as standardized mean differences hedges’ g with their corresponding upper and lower 95% confidence intervals (CI) and prediction intervals (PI), while k indicates sample sizes. Significant results (*p* < 0.05) are indicated by asterisk (*) beside the estimate

Outcome	Biome	k	g	CI	PI
seed density	Mediterranean Forests, Woodlands & Scrub	37	0.78 *	[0.23;1.33]	[-0.92;22.49]
	Temperate Broadleaf & Mixed Forests	12	1.10 *	[0.28;1.92]	[-0.71;2.92]
	Temperate Conifer Forests	6	0.65	[-0.89;2.20]	[-1.58;2.89]
	Temperate Grasslands, Savannas & Shrublands	13	1.84 *	[1.04;2.64]	[0.03;3.64]
	Tropical & Subtropical Dry Broadleaf Forests	8	1.45 *	[0.30;2.61]	[-0.53;3.44]
	Tropical & Subtropical Grasslands, Savannas & Shrublands	4	1.25 *	[0.20;2.31]	[-0.68;3.18]
	Tropical & Subtropical Moist Broadleaf Forests	80	1.37 *	[1.04;1.71]	[-0.28;3.02]
seed richness	Mediterranean Forests, Woodlands & Scrub	3	3.72 *	[1.32;6.11]	[0.33;7.10]
	Temperate Broadleaf & Mixed Forests	2	1.84	[-0.54;4.22]	[-1.54;5.22]
	Tropical & Subtropical Grasslands, Savannas & Shrublands	1	6.30 *	[3.20;9.39]	[2.38;10.21]
	Tropical & Subtropical Moist Broadleaf Forests	47	1.86 *	[1.21;2.51]	[-0.63;4.34]
seedling density	Mediterranean Forests, Woodlands & Scrub	35	1.56 *	[0.82;2.30]	[-0.50;3.61]
	Montane Grasslands & Shrublands	4	0.32	[-1.28;1.92]	[-2.18;2.81]
	Temperate Broadleaf & Mixed Forests	5	0.77	[-0.51;2.05]	[-1.54;3.07]
	Temperate Grasslands, Savannas & Shrublands	6	1.82 *	[0.63;3.01]	[-0.44;4.07]
	Tropical & Subtropical Grasslands, Savannas & Shrublands	1	1.45	[-0.53;3.43]	[-1.30;4.20]
	Tropical & Subtropical Moist Broadleaf Forests	38	0.87 *	[0.39;1.35]	[-1.10;2.85]
seedling richness	Mediterranean Forests, Woodlands & Scrub	3	0.49	[-0.83;1.81]	[-1.48;2.46]
	Montane Grasslands & Shrublands	4	0.20	[-1.11;1.51]	[-1.77;2.17]
	Tropical & Subtropical Grasslands, Savannas & Shrublands	1	2.17 *	0.6	3.74
	Tropical & Subtropical Moist Broadleaf Forests	23	0.79 *	0.28	[-0.76;2.35]

#### Seed richness

We found significant positive effect of perches on seed richness regardless of type (Table 4). We detected between-study variance σ = 5.85 and high heterogeneity I^2^ = 97.99%. Of which, 1.52% and 96.47% were attributed to study level and article level heterogeneity, respectively. Results of the model without modifiers remained positive as well (k = 53, g = 2.16, 95% CI = 1.47–2.84) (Additional File 4 Table 2). Overall, studies reported a higher diversity of seed species under perches compared to areas/traps without perches.

We appraised 32.07% of the 53 studies to have high risk of bias, while the rest (67.92%) were of medium risk of bias (range of g: 1.62–3.64) (Additional File 4 Table 3). Both artificial and natural perches had consensus of positive effects across subgroups, but the effect of semi-natural perches was only apparent in high-risk subgroup. When looking at perches as a group, the effect sizes of general perches for seed richness were not affected by risk of bias. On the other hand, no study was deemed as having high method validity; 58.49% of the studies had low validity while 41.51% had medium validity, and both subgroups showed similar positive effect sizes for perches in general (range of g: 1.86–2.67) (Additional File 4 Table 5). However, when examining different perch types, both natural and artificial perches showed a consensus positive effect across subgroups, but semi-natural perches had mixed effects i.e., significant with low subgroup (*n* = 4) but nonsignificant with medium ones (*n* = 1).

Perches were effective in grasslands (k = 33; g = 2.55, 95% CI = 1.58–3.52) and shrublands (k = 15; g = 1.90, 95% CI = 0.72–3.08) (Fig. 5). The other two matrix types had very few representations (k < 4), but the effect sizes were positive for open cleared lands (k = 2; g = 2.76, 95% CI = 1.10–4.42) and nonsignificant for herbaceous open areas (k = 3, g = 1.19, 95% CI= -0.86-3.24). The model had high I^2^ = 94.58% and between-study variance σ = 1.97 (Additional File 4 Table 7). We could not check the robustness of results because of little to no sampling size for each risk of bias subgroups (e.g., no high-risk studies under grassland) (Additional File 4 Table 8).

This positive effect of perches on seed richness was true across bioregions (Table 5), namely in Tropical & Subtropical Moist Broadleaf Forests, as well as in Mediterranean Forests, Woodlands & Scrub (k = 3), Tropical & Subtropical Grasslands, Savannas & Shrublands (k = 1) but these last two matrix types have very few samples and hence are not conclusive. We did not find significant effect in Temperate Broadleaf & Mixed Forests (k = 2, g = 1.84, 95% CI= -0.54-4.22; note low k). High heterogeneity in the model was detected, with I^2^ = 92.49% (study level: 8.95%; article level: 83.55%) and between-study variance σ = 1.35. We performed a sensitivity analysis using risk of bias scores, but only Tropical & Subtropical Moist Broadleaf Forests had enough samples for comparison, to which it showed robust positive effects (Additional File 4 Table 9).

#### Seedling density

The type of perch affected its effectiveness, as only natural perches showed significant positive effect on seedling density (Table 4). The heterogeneity within our dataset was high at I^2^ = 91.51%, contributed by heterogeneity at study (37.66%) and article level (53.85%), and between-study variance σ = 0.59. When using a three-level model without modifiers, perches in general increased seedling density (k = 89, g = 1.09, 95% CI = 0.75–1.44) (Additional File 4 Table 2).

Out of the 89 studies, 76.40% were of high risk while 23.59% were of medium risk of bias (see Additional File 4 Fig. 1 for criterion scores). Risk of bias and method validity did not influence our effect sizes for perches in general. Examining effects of perch types in detail, natural perches showed positive effects across different risk of bias subgroups, semi-natural perches had consistent non-significant effects, while artificial perches showed significant effects only with the medium-risk subgroup. With regards to the method, 30.34% had high validity, 20.22% with medium, and 49.44% with low validity (Additional File 4 Table 5). All subgroup analysis presented consistent positive results for perch effects in general (range of g for risk of bias: 1.01–1.27; for method validity: 1.02–1.39) (Additional File 4 Tables 3 and 7). Although, when considering different perch types, only natural perches had a consistent positive effect across subgroups, while only medium and high subgroups showed an effect for artificial and semi-natural perches, respectively.

The effect of perches was apparent in grasslands (k = 20, g = 0.99, 95% CI = 0.33–1.65), and shrublands (k = 53, g = 1.3, 95% CI = 0.81–1.80), but not in open cleared lands (k = 23, g = 0.99, 95% CI= -0.01-1.98). Both herbaceous open areas (k = 2, g = 0.70, 95% CI= -1.12-2.53) and urban parks (k = 2, g= -0.10, 95% CI= -1.82-1.61) have very low sample sizes and showed non-significant effect (Fig. 5; Additional File 4 Table 7). All three effects were positive, with greatest effect observed in shrublands and least in grasslands, but the heterogeneity I^2^ was at 91.20% and between-study variance σ = 0.55. Sensitivity analysis based on risk of bias showed consistent positive effect for shrublands and consistent nonsignificant effect for open cleared lands (Additional File 4 Table 8).

**Fig. 6:**
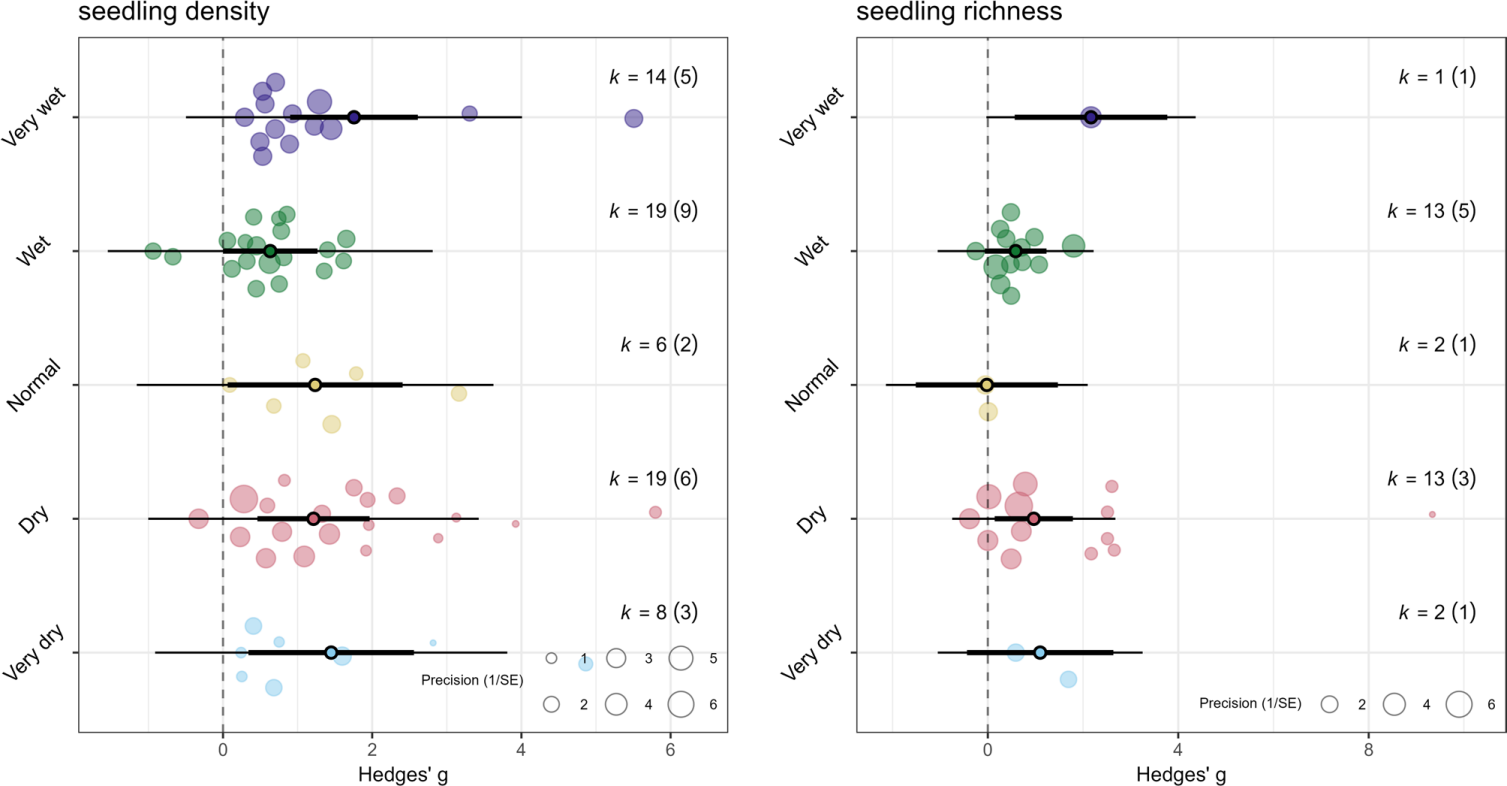
Orchard plot for the standardized mean difference (Hedges’ g) effect sizes on seedling density (left) and richness (right) under different precipitation conditions, showing positive but heterogenous effects for studies conducted during very wet, normal, dry, and very dry years. Precipitation category was assigned based on the annual precipitation levels during the study year in relation to the long-term annual rainfall in the study site from 1981–2023 using CHIRPS dataset [58]. Individual effect sizes (circles) of each study are scaled by their precision (the inverse of standard error). k is the number of effect estimates or the number of studies considered, with the number of articles indicated within parentheses. See model summary statistics in Additional File 4 Table 10

Aside from matrix type, annual precipitation levels during the study showed significant effect as well (Fig. 6, Additional File 4 Table 10). We examined 66 studies for which study year and precipitation data were available and found positive effect of perches during very dry (k = 8, g = 1.45, 95% CI = 0.34–2.56), dry (k = 19, g = 1.21, 95% CI = 0.46–1.96), normal (k = 6, g = 1.23, 95% CI = 0.06–2.41), and very wet (k = 14, g = 1.76, 95% CI = 0.90–2.61) conditions, but none on studies conducted during years with ‘wet’ levels of annual precipitation (k = 19, g = 0.63, 95% CI= -0.00-1.27). The model had high total heterogeneity I^2^ = 87.23%, of which 41.77% was attributed to article and 45.46% to study level, and with between-study variances σ = 0.59.

Biome influenced the effect sizes for seedling density (Table 5). Perches had a significant positive effect in Mediterranean Forests, Woodlands & Scrub, Temperate Grasslands, Savannas & Shrublands, and Tropical & Subtropical Moist Broadleaf Forests (note k = 1), but insignificant effect in Montane Grasslands & Shrublands, Temperate Broadleaf & Mixed Forests, and Tropical & Subtropical Grasslands, Savannas & Shrublands. There was high heterogeneity in the model with I^2^ = 91.05% (study level: 39.90%; article level: 51.16%) and between-study variance σ = 0.54. The positive effect in Tropical & Subtropical Moist Broadleaf Forests was true between risk of bias levels, but other biomes could not be subjected to the sensitivity analysis due to low sample sizes (Additional File 4 Table 9).

#### Seedling richness

As with seedling density, only natural perches were found effective in increasing seedling richness (Table 4). We detected high heterogeneity in the models with I^2^ = 84.64% and between-study variance σ = 0.47. The total heterogeneity was contributed by both article (59.92%) and study level heterogeneities (24.72%). When examining perches in general (i.e., model without moderator), we found significant positive effect of perches on seedling richness (k = 31, g = 0.80, 95% CI = 0.36–1.25); more species of seedlings established in areas with perches compared to areas without perches (Additional File 4 Table 2).

The positive effect of perches on seedling richness appeared to be influenced by risk of bias and method validity. The 31 studies partitioned between high (41.93%) and medium (58.06%) risk of bias (see Additional File 4 Fig. 1 for criterion scores). When considering perches in general, effect sizes were positive and significant for medium-risk studies but nonsignificant for high-risk subset (Additional File 4 Table 3). However, when considering specific perch types, artificial perches had consistent nonsignificant effect across risk levels, while natural perches showed positive effect only for medium-risk studies. For method validity, we classified 29.03% as high, 38.71% as medium, and 32.25% as low validity. The effect sizes were consistently positive across subgroups for perches in general (range of g: 0.34–1.73) (Additional File 4 Table 6). But we found no consensus on the effect of specific perch types; natural perches had positive effects with the medium and high subgroups (i.e., bird-specific) and artificial perches only with the low subgroup.

Matrix type had a significant effect on perch effectiveness (Fig. 5). The effect of perches on seedling richness was positive in grasslands (k = 11, g = 1.40, 95% CI = 0.81–1.99), but nonsignificant in shrublands (k = 14, g = 0.33, 95% CI= -0.26-0.92), herbaceous open areas (k = 2, g = 0.39, 95% CI= -0.92-1.70), and open cleared lands (k = 3, 0.49, 95% CI= -0.62-1.60; but note low sample size). Heterogeneity was high at 78.57% and between-study variance σ of 0.24. The positive effect of perches in grasslands remained positive between two levels of risk of bias (i.e., medium vs. high), while in shrublands it consistently showed nonsignificant effect. We could not implement this sensitivity analysis for other matrix types due to having no representation across risk of bias subgroups (Additional File 4 Table 8).

We examined precipitation as an effect modifier on seedling richness and found positive effect sizes for dry (k = 13, g = 0.96, 95% CI = 0.14–1.79) and nonsignificant effect under wet conditions (k = 13, g = 0.58, 95% CI= -0.60-1.23) (Fig. 6, Additional File 4 Table 10). We had only one sample for very wet conditions and its effect estimate was positive (g = 2.17, 95% CI = 0.56–3.77), while normal and very dry conditions, which were also limited by low sample sizes and hence inconclusive, had nonsignificant and negative effect estimates (Normal: k = 2, g= -0.02, 95% CI= -1.51-1.47; Very dry: k = 2, g = 1.10, 95% CI= -0.44-2.64). The model had between-study variance σ = 0.40 and high total heterogeneity I^2^ = 83.12%. Of which, heterogeneity was attributed mainly to article level (56.95%) and less so at study level (26.17%).

The perch effect on seedling richness varied by biome (Table 5). We found significant positive effect in tropical and subtropical regions, i.e., Tropical & Subtropical Moist Broadleaf Forests and Tropical & Subtropical Grasslands, Savannas & Shrublands (note k = 1), but not in Mediterranean Forests, Woodlands & Scrub and Montane Grasslands & Shrublands, which were each represented by only four studies. The model had high heterogeneity I^2^ = 82.43% (study level: 27.03%; article level: 55.41%) and between-study variance σ = 0.38. We conducted a sensitivity analysis using risk of bias scores, and only Tropical & Subtropical Moist Broadleaf Forests had enough sample size for comparison, which showed consistent positive effects (Additional File 4 Table 9).

#### Sensitivity analysis for publication bias and outlier effects

Using multilevel meta-regression method by Nakagawa et al. (2022) [51], we tested for both publication bias and temporal bias, and results indicated signs of bias for seed richness but not for other outcomes. The slope of the effective sample size was statistically significant (slope= -3.68, 95% CI= -7.14 to -0.23) in the seed richness model (Additional File 4 Table 11; Fig. 3). Upon examination, we found an outlier effect estimate (study 49 with g = 13.59) and once removed, the effect of sample size became nonsignificant, but publication year then showed significant positive effect (slope = 0.13, 95% CI = 0.03–0.23) (Additional File 4 Table 4), indicating temporal bias.

The effect of perches in general remained positive and mostly significant even with removal of influential or outlier studies (Additional File 4 Table 12). Although in the model with perch type as modifiers, we noted a change in the seed richness models, wherein the effect of semi-natural perches became nonsignificant, and in the seedling density models, wherein all perch types had significant effects in contrast to the original model that only identified natural perches as effective (Additional File 4 Table 13). In addition, we also checked the robustness of our model results for seedling density by imputing variances (SD of 0.10, 1.00, 10.00) to the eight studies with zero data, and we found no significant changes to the effect estimates (Additional File 4 Table 14).

### Review limitations

The search strategy for the review was limited to English search strings, which may have excluded regional studies published in other languages. We also had to exclude many studies due to our strict eligibility criteria to ensure the quality of our evidence base, especially so for the meta-analysis, which required mean and variance data. Consequently, the low number of studies with available data prevented us from analysing the effect of distance to forest edge.

About half of the total number of studies included in the review were conducted in just three countries (i.e., Brazil, Spain, and the United States of America), with other countries in the Afrotropic and Palearctic regions and the Indomalaya region at large understudied. In our tests for the effect of modifiers, we had very small sample sizes for certain matrix types, precipitation conditions, and study site biomes, from which we could not draw conclusions.

We also found high heterogeneity in our meta-analysis results. Most of the studies were assessed as having medium and high risk of bias due to potential confounding factors. For example, perches and control sites were placed at varying distances from the forest edge, seed and seedling outcomes could have been affected by predation and herbivory. Moreover, the specificity of the outcomes to being bird-dispersed was unclear, with 33% of the seed-related studies and 48% of the seedling-related studies being assessed as low method validity. Therefore, the outcomes tested here may not all be directly relevant to restoration outcomes, as most studies did not distinguish whether dispersed seeds or seedlings came from the forest or from within the degraded areas. On a related note, the outcomes across studies were measured in different spatial and temporal scales, and this may have contributed to the heterogeneity. For instance, information about the area covered by the study was incomplete, preventing us from examining the effect of scale on the results.

## Review conclusions

Evidence suggests that perches can be used for enhancing natural forest regeneration through increased seed rain and seedling establishment, but effectiveness showed limitations and high variation depending on landscape and bioclimatic factors. Specifically, artificial, semi-natural and natural perches were generally effective in increasing seed density and richness, while natural perches were effective for increasing seedling density and richness. We found robust findings for the positive effect of natural perches on seed density, seed richness, and seedling density, as well as artificial perches on seed richness across risk of bias subgroups, but not for other perch effects. When considering the subset of studies that were highly bird-specific (i.e., high method validity), we found that natural perches were effective for all four outcomes, with strong support for seed density, seed richness, and seedling density, whilst artificial perches had a positive effect for seed richness only and semi-natural perches for seedling density only. The results were clearly influenced by study quality; therefore, results are to be interpreted with caution.

Natural perches, such as tree islets and single standing trees, are known to be important nucleation sites for forest regeneration [76, 77]. Based on our findings, they appear to be more effective over artificial and semi-natural perches, perhaps because they are more natural and have been part of the landscape for a longer time. Foraging by birds can be influenced by spatial memory [78], wherein individuals or groups revisit trees and/or patches which had provided them food in the past, as well as by social learning from peers or other foragers [79, 80]. Tree perches with substantial foliage also offer more cover and hence protection against predators than artificial perches, thus resulting in higher frugivore visits [81].

Placement of the perch in relation to biome and matrix type was also important. As effect sizes can affect the reliability of results, we refrained from concluding about groups with very low samples (k < 5). Perches showed no effects in Temperate Conifer Forest, and this was likely because of limited frugivorous bird assemblage in such habitats that are dominated by wind-dispersed species [75]. For the matrix effect, our findings suggest that, contrary to our initial hypothesis, perches were slightly more effective for seed-related outcomes in less complex habitats (i.e., grasslands) than in more complex ones (i.e., shrublands), although heterogeneity was very high. We initially expected birds to perch in more vegetated habitats than be exposed in an open matrix, but we did not account for the possibility of a ‘dilution effect’ due to availability of other perches. In open landscapes, birds have no other options for perching, thus installing artificial perches can have greater positive impact on seed rain and/or seedling establishment compared to areas with alternative perches (e.g., other trees in the shrubland) [72, 82]. Moreover, generalist bird species, which are usually the most abundant in a degraded landscape, would not have problems using perches in the open areas [68], and so the expected avoidance of matrix was not observed overall.

Even if seed arrival was improved by perches, seeds must germinate and establish as seedlings for forest to regenerate. We found positive effects on seedling density and potentially on richness when using natural perches but not with artificial or semi-natural perches. This agrees with other studies that found no difference in seedling establishment under artificial perches versus control sites [82–84]. The number of seedlings that established was low [85, 86] and the seedling species assemblage was dominated by disturbance tolerant species [82]. Hence, placing artificial perches has limited potential for accelerating forest regeneration. In contrast, studies using natural perches showed promising positive results for both seedling outcomes. This can be due to several factors, including microclimate effects and positive plant interactions. For example, a tree perch can act as a nurse tree that protects seedlings from solar radiation and provides nutrition [87, 88]. We also found that perch effectiveness varied with precipitation conditions, but results did not seem to follow a pattern (i.e., positive effect in both extremes). Our results suggest that either the rainfall effect is negligible in this context or difficult to establish based on published data due to high heterogeneity and low sample sizes. Although, one possible explanation for the observed effects on seedling density is the buffering effect of trees against rainfall intensities during ‘very wet’ conditions [89] and drought during ‘very dry’ conditions [90]. We also expected for ‘wet’ conditions to have a significant positive effect on observed relationships between seedling related outcomes and perches, because germination and seedling growth are dependent on rain for soil moisture [41]. As climate change progresses, we need to better understand these climatic impacts on restoration activities to enhance climate change resilience [91].

In the matrix, seeds experience several barriers to establishment [83, 92], including but not limited to, seed predation [93], exposure to direct sun and rain, lack of soil nutrients, and competition with grasses and weeds [84, 94]. Therefore, restoration programs using perches would benefit from additional treatments for increased seedling establishment. Elgar et al. (2014), for example, used chemical treatment to suppress pasture grasses under perches and found significantly more native woody seedling in these plots compared to untreated ones [95]; however, effectiveness of weed control as an ANR strategy varies by context (e.g., degradation status, time, method used) [96]. Other studies combined soil amendment with perches. They found that adding soil nutrients did not increase seedling establishment, but preserving the existing or pioneer vegetation (e.g., plants and organic material but not weeds) can facilitate seedling establishment, likely through moderating adverse microclimate conditions [97]. Other techniques, such as topsoil transposition, can also be explored to enhance the positive effect of perches [98].

The data examined was likely biased toward studies with substantial results. Almeida et al. (2016) followed up on a perch experiment on seed rain to determine subsequent seedling establishment in a two-year period [83]. They found very low number of seedlings establishing in their plots (< 10 seedlings) compared to the number of seeds arriving (25,755 seeds); having very few data prevented them from conducting statistical tests. Moreover, in eight other studies, not a single seedling grew under perches and control plots [94, 99–101]. One of the possible explanations for the lack of data is the length of the study period [99]. Some seed species require more time and/or specific environmental triggers to germinate, and the study period may not have been long enough to detect seedling establishment. Also, for studies that were conducted within a year, long-term seedling survival would not have been accounted for. For example, juniper seedlings in one study conducted in the USA were abundant during the first spring and summer after dispersal when the study was conducted [102], but saplings (> 50 cm in height) were rare in the area, suggesting low survival rates of seedlings. Consequently, the apparent positive effect of perches observed in these studies may have been short-lived.

We found high heterogeneity in our effect sizes, which is common in ecological meta-analyses. Senior et al. (2016) reviewed 700 ecological and evolutionary meta-analyses and reported a mean *I*^2^ of around 92% [103], which was not far from the observed *I*^2^ (84–91%) in our meta-analysis. And although the prediction intervals of the effect of different perch types on the outcomes included negative Hedges' g values, which meant that in some cases the use of perch can have null effect on the outcomes, we believe that such is the context-dependent nature of most conservation interventions. In reality, it is very unlikely to find an intervention that will always be reported as successful. We found that perch type, matrix type, biome, and precipitation variation impacted the effect sizes, and we expect that other unaccounted factors, such as differences in distance to forest and perch design/specifics, and interaction effects of covariates were driving the unexplained heterogeneity. The slight variation in the significance of effects found in our sensitivity analysis also showed that risk of bias and method validity (i.e., how bird-specific the species were) likely affected our findings. Notably, all our studies were classified as having either an overall high or medium risk of bias, which was heavily influenced by criteria related to blinding (e.g., Criterion 2.2 & 4.1 & 5.1), a research strategy uncommon in ecological studies [104]. CEE Critical Appraisal tool also applies a simple approach to assigning an overall risk of bias score (e.g., overall high-risk to a study that scored high in at least one of the seven criteria, medium if it scored at least one medium but no high, and so on), and thus overall scores can be heavily impacted by a single criterion. We observed that effect sizes were generally higher in the high-risk subgroup compared to medium-risk, suggesting that biases can inflate the effect sizes. In addition, studies with small sample sizes and high risk of bias had relatively high positive effect estimates (see Additional File 4 Fig. 3), which is indicative of effect overestimation in smaller studies with low study quality [105]. This highlights the need for ecological researchers to mitigate and control for study biases, as these can make research findings unreliable. On top of these biases occurring at the study stage, biases can occur at publication stage. We found higher effect sizes in studies published in more recent years compared to earlier ones, but we could not find an underlying methodological and contextual factor to explain the trend (see Additional File 4 Fig. 2 for plot exploring effect of perch type, biomes, etc.). Thus, it is likely a product of the high heterogeneity in our studies, but we also do not discount the possibility of publication bias.

### Implications for policy/management

To increase seed rain in the degraded landscapes, all three perch types can be used. However, only natural perches showed robust effects of increasing seedling establishment. We recommend that restoration activities should preserve existing natural perches and establish more perches in areas lacking these natural features. Given that artificial perches are relatively easier and faster to install, we suggest using artificial/semi-natural perches to increase seed rain and employ other interventions to enhance seedling establishment [18, 95], such as weeding, watering, and soil amelioration, but this might entail more cost and effort.

Location and landscape context are also important considerations to decide whether perches will work or not. Perches can only be effective if birds are the main dispersers of plants in the area, hence they are useful in restoring degraded lands near tropical and subtropical moist and dry forests, but not in conifer forests, which consist of plants that are mainly wind-dispersed. Perches also seem to provide maximum positive impact in open areas (i.e., grasslands, agricultural fields), compared to habitats with trees and shrubs that can already act as natural perching sites.

Lastly, like with other restoration programs, we recommend that perches should be monitored at regular time periods, especially given the many confounding variables that may influence their effectiveness [106]. For one, perches cannot discriminate between plant species that they receive; it is possible that unwanted species (e.g., invasive, non-forest, dominant species) are benefiting instead of target species (e.g., forest species) for restoration. Hence, monitoring is crucial for the development and reassessment of the overall restoration program.

### Implications for research

Based on our narrative synthesis, the efficacy of perches in general appeared to be unaffected by perch distance to the forest edge, as only a few studies reported positive or negative results. Distance to edge can easily be confounded by several factors that can affect different aspects of plant recruitment [95]. For instance, seed rain for zoochorous plant species is ultimately regulated by the frugivore community. Negative effects, wherein perches farther from the forest edge received less seed inputs, likely occur because of birds exhibiting preferences to stay near the forest than to venture into the more open and possibly high-risk matrix [34, 38, 108]. In contrast, when generalist species dominate the frugivore community, then the effect of distance would not be as pronounced as these birds can move between and utilize both matrix and forest habitats [109]. Moreover, the distance effect can change depending on the range of distances covered in the analysis and interact with matrix habitat structure [33]. We were not able to explore this relationship in the meta-analysis due to insufficient data. We call for more perch studies in relation to varying distances to be conducted and ideally accompanied by survey of the frugivore community and the matrix habitat to be able to discern underlying relationships.

We noted relatively low numbers of studies conducted in Australasia, Oceania, and Indomalaya regions. This is to be expected for Australasia, as the region includes wide expanses of deserts and xeric shrublands, and Oceania holds only a small portion of the world’s forests due to its size [60]. However, the Indomalaya region consists mostly of forest habitats, which are threatened by deforestation [110] and would thus benefit from more research on forest restoration. Moreover, the spatial coverage of these regions was limited to a few countries. Although Afrotropic and Palearctic regions have relatively good representation in terms of the number of studies, many countries were not represented; there was a clear concentration of research efforts in certain countries like Spain and Uganda. In terms of forest biome representation in perch studies, Temperate Forests (i.e., conifer, broadleaf & mixed) and Tropical & Subtropical Dry Broadleaf Forests received far less attention compared to wet forests [111]. Dry forests account for about 40% of all tropical forests and face great risks from anthropogenic and natural changes [112]. We recommend that more perch studies be conducted in the dry forest biome, where birds are one of the major seed dispersers [24], to inform forest conservation.

Lastly, it would be interesting to update our systematic review and meta-analysis to include studies in other languages (e.g., Portuguese, Chinese, French) in the future. As per our eligibility criteria, we have excluded articles that were not in English. Our review missed relevant studies, such as those that examined both artificial perches and soil translocation as nucleation techniques in Brazil because the text was written in Portuguese [113]. We have also excluded a lot of studies (63 studies) because the mean and variance data were unavailable for the control and/or intervention. As a result of this, we could not analyse the effect of distance to forest edge as a moderator. Future studies can explore the use of other weighting methods that do not rely on variance estimates, such as adjusted variance and sample size, which reportedly can provide more accurate meta-estimates [114]. We also encourage researchers to adapt open science practices (e.g., open data, open code) [115]. Finally, on top of data sharing, good reporting standards are crucial. We had a couple of studies which reported variances but failed to state whether it was standard error or standard deviation.

Similar to those used in the medical sciences, we encourage the development of reporting guidelines and checklists for ecological studies. Journals can also help improve reporting standards. For example, the journal *Ecological Letters* provides a checklist to guide authors in reporting details of their study methodology [116], and this can be expanded to cover reporting of the results (e.g., variances, test statistics).

## Electronic supplementary material

Below is the link to the electronic supplementary material.

**Supplementary Material 1**: **Additional File 1**: AF1_Systematic Review Stakeholder Survey. The questionnaire used for the stakeholder survey.

**Supplementary Material 2**: **Additional File 2**: AF2_Final_search_string_and_fulltext_exclusion. The final search strings used for the eight databases and the resulting number of search hits, and the list of articles excluded at full-text and critical appraisal stages with reasons.

**Supplementary Material 3**: **Additional File 3**: AF3_Dataset. The dataset used for the narrative synthesis and meta-analysis, extracted from the eligible articles.

**Supplementary Material 4**: **Additional File 4**: AF4_meta-analysis_supplementary. The supporting documents for the meta-analysis, including results of meta-regression analysis and sensitivity analysis.

**Supplementary Material 5**: **Additional File 5**: AF5_ROSES for Systematic Review Reports. The ROSES checklist for systematic review report.

**Supplementary Material 6**: **Additional File 6**: AF6_PRISMA Abstract Checklist. The PRISMA checklist for abstract.

**Supplementary Material 7**: **Additional File 7**: AF7_PRISMA Main Checklist. The PRISMA checklist for main manuscript.

**Supplementary Material 8**: **Additional File 8**: AF8_Critical_Appraisal_dataset. The dataset containing the risk of bias and method validity assessment for each eligible study.

## Data Availability

Data is provided within the manuscript or additional files.
