# Supplementary material for: Effectiveness of perches in promoting bird-mediated seed dispersal for natural forest regeneration: a systematic review
Source: Environ Evid. 2025 Jun 14;14:10. doi: 10.1186/s13750-025-00363-8 (PMC12166613; doi:10.1186/s13750-025-00363-8)
Supplement: Supplementary file 2 — Supplementary Material 2: Additional File 2: AF2_Final_search_string_and_fulltext_exclusion. The final search strings used for the eight databases and the resulting number of search hits, and the list of articles excluded at full-text and critical appraisal stages with reasons. [file 13750_2025_363_MOESM2_ESM.docx]

**Effectiveness of perches in promoting bird-mediated seed dispersal for natural forest regeneration: A systematic review**

**Additional File 2.** The final search strings used for the eight databases and the resulting number of search hits, and the list of articles excluded at full-text and critical appraisal stages with reasons. This file contains the following tables:

Table 1. The final search strings used for the eight databases and the resulting number of search hits.

Table 2. List of articles excluded at full-text screening stage with reasons.

Table 3. List of excluded articles after critical appraisal.

**Table 1. The final search strings used for the eight databases and the resulting number of search hits.**

| **Database/ citation indexing service** | **Institutional subscription** | **Search fields** | **Search string** | **Search hits** | **Search date** |
| --- | --- | --- | --- | --- | --- |
| Web of Science Core Collection: Science Citation Index Expanded (SCI-EXPANDED)--1970-present Social Sciences Citation Index (SSCI)--1970-present Arts & Humanities Citation Index (AHCI)--1975-present Conference Proceedings Citation Index – Science (CPCI-S)--1990-present Conference Proceedings Citation Index – Social Science & Humanities (CPCI-SSH)--1990-present Emerging Sources Citation Index (ESCI)--2015-present | Newcastle University | Topic (includes title, abstract, author keywords, and keywords plus) | TS= (( bird* OR avian OR aves OR disperse*) AND ( palm* OR fruit* OR perch* OR "artificial perch*" OR roost* OR nucleation* OR nuclei OR "tree isl*" OR "woodland isl*" OR "habitat isl*" OR "remnant tree*" OR "isolated tree*" OR "single tree*" OR shrub* OR wire* OR post* OR scrub*) AND ( "seed dispers*" OR "seed rain*" OR seedling* OR regenerat*) AND (forest* OR woodl*)) | **2,873** | 07-Sep-23 |
| Zoological Record | Newcastle University | Topic (includes title, book title, abstract, broad terms, descriptors data, super taxa, systematics, taxa notes) | TS= (( bird* OR avian OR aves OR disperse*) AND ( palm* OR fruit* OR perch* OR "artificial perch*" OR roost* OR nucleation* OR nuclei OR "tree isl*" OR "woodland isl*" OR "habitat isl*" OR "remnant tree*" OR "isolated tree*" OR "single tree*" OR shrub* OR wire* OR post* OR scrub*) AND ( "seed dispers*" OR "seed rain*" OR seedling* OR regenerat*) AND (forest* OR woodl*)) | **542** | 07-Sep-23 |
| SciELO Citation Index | Newcastle University | Topic (includes title, abstract, author keywords) | TS= (( bird* OR avian OR aves OR disperse*) AND ( palm* OR fruit* OR perch* OR "artificial perch*" OR roost* OR nucleation* OR nuclei OR "tree isl*" OR "woodland isl*" OR "habitat isl*" OR "remnant tree*" OR "isolated tree*" OR "single tree*" OR shrub* OR wire* OR post* OR scrub*) AND ( "seed dispers*" OR "seed rain*" OR seedling* OR regenerat*) AND (forest* OR woodl*)) | **123** | 07-Sep-23 |
| Scopus | Newcastle University | Article Title, Abstract, Keywords | TITLE-ABS-KEY ( ( bird* OR avian OR aves OR disperse* ) AND ( palm* OR fruit* OR perch* OR "artificial perch*" OR roost* OR nucleation* OR nuclei OR "tree isl*" OR "woodland isl*" OR "habitat isl*" OR "remnant tree*" OR "isolated tree*" OR "single tree*" OR shrub* OR wire* OR post* OR scrub* ) AND ( "seed dispers*" OR "seed rain*" OR seedling* OR regenerat* ) AND (forest* OR woodl*)) | **2,302** | 07-Sep-23 |
| CAB Abstracts | Newcastle University | WOK Free-Text index (English Item Title, Original Item Title, Source Abstract, CABICODE Names, Descriptors, Organism Descriptors, Geographic Location, Identifiers, Broad Terms) | ( (bird* OR avian OR aves OR disperse*) ) AND ( (palm* OR fruit* OR perch* OR "artificial perch*" OR roost* OR nucleation* OR nuclei OR "tree isl*" OR "woodland isl*" OR "habitat isl*" OR "remnant tree*" OR "isolated tree*" OR "single tree*" OR shrub* OR wire* OR post* OR scrub*) ) AND ( ("seed dispers*" OR "seed rain*" OR seedling* OR regenerat*) ) AND ( (forest* OR woodl*) ) | **3,162** | 07-Sep-23 |
| ProQuest Natural Science Collection (Coverage: 1946 - current) | Newcastle University | Title, Abstract, Keywords | AB,TI,IF(( bird* OR avian OR aves OR disperse*) AND ( palm* OR fruit* OR perch* OR "artificial perch*" OR roost* OR nucleation* OR nuclei OR "tree isl*" OR "woodland isl*" OR "habitat isl*" OR "remnant tree*" OR "isolated tree*" OR "single tree*" OR shrub* OR wire* OR post* OR scrub*) AND ( "seed dispers*" OR "seed rain*" OR seedling* OR regenerat*) AND (forest* OR woodl*)) | **2,946** | 07-Sep-23 |
| Conservation Evidence | Open Access | NA | search studies using keyword "perch" and under category "birds" | **32** | 07-Sep-23 |
| Google Scholar | Open Access | NA | (bird) AND (perch* OR tree OR shrub OR wire* OR post*) AND (seed dispersal OR seed rain OR seedling* OR regenerat*) AND (forest* OR woodl*) | **200**  Sorted by relevance | 08-Sep-23 |
|  |  |  | Bird AND perch AND seed dispersal | **200** | 08-Sep-23 |
|  |  |  | TOTAL | **12,269** |  |

**Table 2. List of articles excluded at full-text screening stage with reasons.**

| **Study title** | **year** | **journal** | **authors** | **reason** |
| --- | --- | --- | --- | --- |
| Aggregated recruitment patterns under adult crowns in Photinia glabra, a bird-dispersed tree species | 2017 | Plant Species Biology | Kuge, A & Hirayama, K | no perch |
| Aggregated seed dispersal by wreathed hornbills at a roost site in a moist evergreen forest of Thailand | 2008 | Ecological Research | Kitamura, S., Yumoto, T., Noma, N., Chuailua, P., Maruhashi, T., Wohandee, P., & Poonswad, P. | no perch |
| Annual changes in a bird assembly on artificial perches: Implications for ecological restoration in a subtropical agroecosystem | 2016 | Biota Neotropica | Vogel, H. F., Spotswood, E., Campos, J. B., & Bechara, F. C. | no data |
| Applied nucleation facilitates tropical forest recovery: Lessons learned from a 15-year study | 2020 | Journal of Applied Ecology | Holl, K. D., Reid, J. L., Cole, R. J., Oviedo-Brenes, F., Rosales, J. A., & Zahawi, R. A. | no perch |
| Architecture of remnant trees influences native woody plant recruitment in abandoned Hawaiian pastures | 2021 | Plant Ecology | Rehm, E. M., Yelenik, S. G., Smith, M. P., & D’Antonio, C. M. | no control |
| Arrested succession in pastures hinders regeneration of Tropandean forests and shreds mountain landscapes | 1997 | Environmental Conservation | Sarmiento, F. O. | no control |
| Arrival ≠ Survival | 2013 | Restoration Ecology | Reid, J. L., & Holl, K. D. | no data |
| Artificial Perches and Windrowing of Brushwood To Recovery of Degraded Area in the Semiarid of Paraíba State, Brazil | 2015 | Nativa | Silveira, L. D., Souto, J. S., Damasceno, M. M., Mucida, D. P., & Pereira, I. M. | foreign language |
| Artificial perches as a nucleation technique for restoration of a riparian environment: characterization of the seed rain and of natural regeneration | 2010 | Biotemas | Tomazi, A. L., Zimmermann, C. E., & Laps, R. R. | foreign language |
| Artificial perches promote vegetation restoration | 2016 | Plant Ecology | Guidetti, B. Y., Amico, G. C., Dardanelli, S., & Rodriguez-Cabal, M. A. | no data |
| Artificial roosts as seed dispersal nuclei in a cerrado area in Triângulo Mineiro, Brazil | 2016 | Bioscience Journal | Ferreira, G. Â., & Melo, C. D. | no control |
| Avian frugivory and seed dispersal of a large fruited tree in an Indian moist deciduous forest | 2015 | Acta Oecologica | Chatterjee, S., & Basu, P. | no data |
| Avian seed dispersal and seedling distribution of the endangered tree species, Taxus chinensis, in patchy habitats | 2015 | Plant Ecology and Diversity | Li, N., Bai, B., Wang, Z., Luo, F., Lu, X. Z., & Lu, C. H. | no control |
| Avian Seed Dispersal on Virginia Barrier Islands: Potential Influence on Vegetation Community Structure and Patch Dynamics | 2010 | The American Midland Naturalist Journal | Shiflett, S. A., & Young, D. R. | no control |
| Barriers to forest regeneration of deforested and abandoned land in Panama | 2005 | Journal of Applied Ecology | Hooper, E., Legendre, P., & Condit, R. | no perch |
| Barriers to Lowland Tropical Forest Restoration in the Sierra Nevada de Santa Marta, Colombia | 1994 | Restoration Ecology | Aide, T. M., & Cavelier, J. | no control |
| Bat- and bird-generated seed rains at isolated trees in pastures in a tropical rainforest | 2000 | Conservation Biology | Galindo-Gonzalez, J, Guevara, S & Sosa, V J | no control |
| Biological and environmental effects on fine-scale seed dispersal of an invasive tree in a secondary subtropical forest | 2018 | Biological Invasions | Powell, P. A., & Aráoz, E. | no control |
| Bird and ant synergy increases the seed dispersal effectiveness of an ornithochoric shrub | 2016 | Oecologia | Camargo, P. H., Martins, M. M., Feitosa, R. M., & Christianini, A. V. | no perch |
| Bird assemblages in isolated Ficus trees in Kenyan farmland | 2006 | Journal of Tropical Ecology | Eshiamwata, G. W., Berens, D. G., Bleher, B., Dean, W. R. J., & Böhning-Gaese, K. | no data |
| Bird communities and seedling recruitment in restoring seasonally dry forest using the framework species method in Northern Thailand | 2009 | New Forests | Wydhayagarn, C., Elliott, S., & Wangpakapattanawong, P. | no control |
| Bird-mediated seed dispersal of fleshy fruits of mediterranean shrubs in semiarid forest patches: the role of Pinus halepensis Miller trees as seed receptors | 2014 | Plant Ecology | Zapata, V. M., Robledano, F., Ramos, V., & Martínez-López, V. | no control |
| Birds and small mammals in kanuka (Kunzea ericoides) and gorse (Ulex europaeus) scrub and the resulting seed rain and seedling dynamics | 2002 | New Zealand Journal of Ecology | Williams, P. A. & Karl, B. J. | no control |
| Bottlenecks for plant recruitment in woodland remnants: An ornithochorous shrub in a Mediterranean 'relictual' landscape | 2012 | Perspectives in Plant Ecology, Evolution and Systematics | González-Varo, J. P., Nora, S., & Aparicio, A. | no perch |
| Can salvage logging affect seed dispersal by birds into burned forests? | 2009 | Acta Oecologica | Rost, J., Pons, P. & Bas, J. M. | no control |
| Canopy asymmetry in solitary Diphysa americana trees: wind and landscape on the Mexican coast | 2019 | Journal of Coastal Conservation | Ramírez-Pinero, M., Lira-Noriega, A., & Guevara, S. | no perch |
| Changes in the fruiting landscape relax restrictions on endozoochorous tree dispersal into deforested lands | 2015 | Applied Vegetation Science | Martínez, D., & García, D. | no perch |
| Colonization of agricultural windbreaks by forest trees: effects of connectivity and remnant trees | 2000 | Ecological Applications | Harvey, C. A. | no control |
| Colonization of Cape fynbos communities by forest species | 1992 | Forest Ecology and Management | Manders, P T & Richardson, D M | no control |
| Comparison of nucleation techniques effectiveness for degraded area restoring in southern Brazil | 2013 | Floresta | Marcuzzo, S. B., Ganade, G., Araújo, M. M., & Muniz, M. F. B. | foreign language |
| Composition and clumping of seeds deposited by frugivorous birds varies between forest microsites | 2011 | Oikos | Blendinger, P. G., Blake, J. G., & Loiselle, B. A. | no control |
| Connecting fruit production to seedling establishment in two co-occurring Miconia species: Consequences of seed dispersal by birds in upper Amazonia | 2011 | Oecologia | Blendinger, P G, Blake, J G & Loiselle, B A | no control |
| Consequences of frugivore diversity for seed dispersal, seedling establishment and the spatial pattern of seedlings and trees | 2001 | Oecologia | Bleher, B., & Böhning-Gaese, K. | no control |
| Conserving the diversity of ecological interactions: the role of two threatened macaw species as legitimate dispersers of "megafaunal" fruits | 2020 | Diversity | Tella, J. L., Hiraldo, F., Pacífico, E., Díaz-Luque, J. A., Dénes, F. V., Fontoura, F. M., ... & Blanco, G. | no control |
| Contagious dispersal of seeds of synchronously fruiting species beneath invasive and native fleshy-fruited trees | 2011 | Austral Ecology | White, E & Vivian-Smith, G | no control |
| Contagious seed dispersal and the spread of avian-dispersed exotic plants | 2015 | Biological Invasions | Bonilla, N O & Pringle, E G | no control |
| Contagious seed dispersal beneath heterospecific fruiting trees and its consequences | 2004 | Oikos | Kwit, C and Levey, D J & Greenberg, C H | no control |
| Contribution of artificial perches to seed dispersal and its application to forest restoration | 2014 | Ciencia Florestal | Dias, C R and Umetsu, F & Breier, T B | foreign language |
| Contribution of nucleation techniques to plant establishment in restoration projects: an integrative review and meta-analysis | 2023 | Restoration Ecology | de Oliveira Bahia, T., Martins, C., Antonini, Y., & Cornelissen, T. | no data |
| Contribution of the wildlife in ecological restoration projects in Brazil [Contribuicao da fauna silvestre em projetos de restauracao ecologica no Brasil] | 2012 | Pesquisa Florestal Brasileira | Campos, W. H., Neto, A. M., Peixoto, H. J. C., Godinho, L. B., & Silva, E. | foreign language |
| Crossing the threshold: Invasive grasses inhibit forest restoration on Hawaiian islands | 2023 | ECOLOGICAL APPLICATIONS | Rehm, E. M., D'Antonio, C., & Yelenik, S. | no control |
| Differential contribution of frugivorous birds to dispersal patterns of the endangered Chinese yew (Taxus chinensis) | 2015 | Scientific Reports | Li, N., Fang, S. B., Li, X. H., An, S. Q., & Lu, C. H. | no control |
| Directed seed dispersal by bellbirds in a tropical cloud forest | 1998 | Proceedings of the National Academy of Sciences of the United States of America | Wenny, D G & Levey, D J | no perch |
| Dispersal ecology of Phoenix sylvestris Roxb | 1989 | Environment & Ecology | Mishra, R M & Singh, S K | missing |
| Dispersal of Acacia cyclops by birds | 1981 | Oecologia | Glyphis, J. P., Milton, S. J., & Siegfried, W. R. | no control |
| Dispersal of eastern red cedar (Juniperus virginiana) into pastures: an overview | 1987 | Canadian Journal of Botany | Holthuijzen, A. M., Sharik, T. L., & Fraser, J. D. | no control |
| Dispersal of invasive Phytolacca americana seeds by birds in an urban garden in China | 2017 | Integrative Zoology | Li, N., Yang, W., Fang, S., Li, X., Liu, Z., Leng, X., & An, S. | no control |
| Dispersal of remnant endangered trees in a fragmented and disturbed forest by frugivorous birds | 2017 | Journal of Plant Research | Li, N., Bai, B., Li, X. H., An, S. Q., & Lu, C. H. | no control |
| Dispersal of Spanish juniper Juniperus thurifera by birds and mammals in a fragmented landscape | 1999 | Ecography | Santos, T., Tellería, J. L., & Virgós, E. | no control |
| Dispersal Patterns of One-Seed Juniper Seeds Contained in Mammal Scats and Bird Pellets | 2022 | Forests | Stricklan, D., Cibils, A. F., Saud, P., Steiner, R. L., McIntosh, M. M., Ganguli, A. C., ... & Faist, A. M. | no control |
| Do birds bias measurements of seed rain? | 2012 | Journal of Tropical Ecology | Reid, J. L., Katsuki, K. N., & Holl, K. D. | no control |
| Do Coarser Gap Mosaics in Conifer Plantations Induce More Seed Dispersal by Birds? Temporal Changes during 12 Years after Gap Creation | 2019 | Forests | Takano, T., Kominami, Y., & Mizunaga, H. | no perch |
| Early fate of Myristica hypargyraea seeds dispersed by Ducula pacifica in Tonga, Western Polynesia | 2005 | Austral Ecology | Meehan, H. J., McConkey, K. R., & Drake, D. R. | no control |
| Early Successional Patterns and Potential Facilitation of Woody Plant Colonization by Rotting Logs in Premontane Costa Rican Pastures | 2000 | Restoration Ecology | Peterson, C. J., & Haines, B. L. | no perch |
| Ecological significance of bird perches on the restoration of forest vegetation | 2009 | Acta Ecologica Sinica | Li, X. H. | foreign language |
| Effect of artificial perches on seed dispersal by birds [Efeito de poleiros artificiais na dispersão de sementes por aves] | 2000 | Revista Árvore | Melo, V A, Griffith J J, Marco, P Jr., Silva, E, Souza, A L, Guedes, M C and Ozório, T F | foreign language |
| Effect of frugivorous birds on the establishment of a naturally regenerating population of Chinese yew in ex situ conservation | 2008 | Integrative Zoology | Lu, C., Zhu, Q., & Deng, Q. | no perch |
| Effectiveness of three turacos as seed dispersers in a tropical montane forest | 1997 | Oecologia | Sun, C., Ives, A. R., Kraeuter, H. J., & Moermond, T. C. | no perch |
| Effects of bird traits on seed dispersal of endangered Taxus chinensis (Pilger) Rehd. with ex-situ and in-situ conservation | 2019 | Forests | Li, N., Wang, Z., Li, X. H., Yi, X. F., Yan, C., Lu, C. H., & Chen, S. C. | no control |
| Effects of forest fragmentation on seed dispersal and seedling establishment in ornithochorous trees | 2010 | Conservation Biology | Herrera, J. M., & Garcia, D. | no perch |
| Effects of palatability and dispersal mode on spatial patterns of trees in oldfields | 1999 | NCASI Technical Bulletin | Myster, R W & Pickett, S T A | no perch |
| Empirical evaluation of directed dispersal and density-dependent effects across successive recruitment phases | 2012 | Journal of Ecology | Spiegel, O., & Nathan, R | no perch |
| Engaging birds in vegetation restoration after Elwha dam removal | 2013 | Ecological Restoration | McLaughlin, J F | no data |
| Enhancing bird-mediated seed dispersal to increase rainforest regeneration in disused pasture – A restoration experiment | 2021 | Forest Ecology and Management | Freeman, A. N., Freebody, K., Montenero, M., Moran, C., Shoo, L. P., & Catterall, C. P. | no control |
| Exclusive frugivory and seed dispersal of Rhamnus alaternus in the bird breeding season | 2006 | Plant Ecology | Bas, J. M., Pons, P., & Gomez, C. | no control |
| Exotic guavas are foci of forest regeneration in Kenyan farmland | 2008 | Biotropica | Berens, D. G., Farwig, N., Schaab, G., & Böhning-Gaese, K. | no control |
| Facilitation by herbivore-mediated nurse plants in a threatened tree,Taxus baccata:local effects and landscape level consistency | 2003 | Ecography | García, D., & Ramón Obeso, J | no perch |
| Factors affecting seed rain beneath fleshy-fruited plants | 2004 | Plant Ecology | Takahashi, K., & Kamitani, T. | no control |
| Feeding behavior, bird visitation, and seed dispersal in Guarea macrophylla and Trichilia quadrijuga (Meliaceae) | 2013 | Ornitologia Neotropical | Prado, F. A. | no perch |
| Ficus cestrifiolia (Moraceae) as a natural roost: a strategy in restoration projects for degraded areas | 2008 | Natureza & Conservação | Krieck, C. A., Fink, D., & Zimmermann, C. E. | foreign language |
| Ficus natalensis facilitates the establishment of a montane rain-forest tree in south-east African tropical woodlands | 2014 | Journal of Tropical Ecology | Fujita, T., & Yamashina, C. | wrong location |
| Ficus seed shadows in a Bornean rain forest | 1996 | Oecologia | Laman, T. G. | no control |
| Fleshing out facilitation - reframing interaction networks beyond top-down versus bottom-up | 2016 | New Phytologist | Watson, D. M. | no data |
| Foraging and dispersal of birds on Ficus microcarpa fruit in urban green space | 2023 | Journal of Anhui Agricultural Sciences | Wang, G. H., Tang, C. B., & Wei, L. J. | foreign language |
| Forest and woodland restoration for wildlife | 2017 | Biodiversity | Weir, J | no perch |
| Forest fragmentation severs mutualism between seed dispersers and an endemic African tree | 2003 | Proceedings of the National Academy of Sciences of the United States of America | Cordeiro, N J & Howe, H F | no control |
| Formación del banco de semillas durante la revegetación temprana de áreas afectadas por la minería en un bosque pluvial tropical del Chocó, Colombia | 2017 | Revista de Biologia Tropical | Valois-Cuesta, H., Martínez-Ruiz, C., & Urrutia-Rivas, Y. | foreign language |
| From the matrix to roadsides and beyond: the role of isolated paddock trees as dispersal points for invasion | 2014 | Diversity and Distributions | Coulson, C., Spooner, P. G., Lunt, I. D., & Watson, S. J. | no control |
| Frugivore biodiversity and complementarity in interaction networks enhance landscape-scale seed dispersal function | 2018 | Functional Ecology | García, D., Donoso, I., & Rodríguez‐Pérez, J. | no data |
| Frugivore diversity increases evenness in the seed rain on deforested tropical landscapes | 2022 | Oikos | Camargo, P.H.S.A., Carlo, T A, Brancalion, P H S, & Pizo, M A | no control |
| Frugivores bias seed-adult tree associations through nonrandom seed dispersal: A phylogenetic approach | 2016 | Ecology | Razafindratsima, O H & Dunham, A E | no bird |
| Frugivorous birds influence the spatial organization of tropical forests through the generation of seedling recruitment foci under zoochoric trees | 2017 | Acta Oecologica | Trolliet, F., Forget, P. M., Doucet, J. L., Gillet, J. F., & Hambuckers, A. | no control |
| Frugivory and habitat use by fruit-eating birds in a fragmented landscape of southeast Brazil | 2004 | Ornitologia Neotropical | Pizo, M. A., & dos Santos, B. T. | no perch |
| Frugivory and seed dispersal in Cymbopetalum baillonii (Annonaceae) at Los Tuxtlas, Mexico | 1988 | Journal of Tropical Ecology | Coates-Estrada, R., & Estrada, A. | no perch |
| Frugivory and seed dispersal of Podocarpus nubigena in Chiloe , Chile | 1996 | Revista chilena de historia natural | Willson, M. F., Sabag, C., Figueroa, J., & Armesto, J. J. | no control |
| Frugivory, Post-feeding Flights of Frugivorous Birds and the Movement of Seeds in a Brazilian Fragmented Landscape | 2011 | Biotropica | Pizo, M. A. | no perch |
| Fruiting and frugivores at a strangler fig in the tropical rain forest of Los Tuxtlas, Mexico | 1986 | Journal of Tropical Ecology | Coates-Estrada, R., & Estrada, A. | no perch |
| Fruiting trees as dispersal foci in a semi-deciduous tropical forest | 2004 | Oecologia | Clark, C J, Poulsen, J R, Connor, E F & Parker, V T | no perch |
| Functional complementarity of seed dispersal services provided by birds and mammals in an alpine ecosystem | 2022 | Journal of Ecology | García-Rodríguez, A., Albrecht, J., Farwig, N., Frydryszak, D., Parres, A., Schabo, D. G., & Selva, N. | no perch |
| Functional importance of bird-dispersed habitat for the early recruitment of Taxus chinensis in a fragmented forest | 2022 | Acta Oecologica | Wang, Z., Gao, S., Huang, X., Zhang, S., & Li, N. | no perch |
| Gardeners of the forest: hornbills govern the spatial distribution of large seeds | 2021 | Journal of Avian Biology | Naniwadekar, R., Mishra, C., Isvaran, K., & Datta, A. | no perch |
| How does avian seed dispersal shape the structure of early successional tropical forests? | 2019 | Functional Ecology | González-Castro, A., Yang, S., & Carlo, T. A. | no control |
| Impacts of hunting on seed dispersal in a Central African tropical forest | 2008 | ProQuest Dissertations and Theses | Wang, B. C. | wrong location |
| Importance of Forests Outside Protected Area Networks for Large-Seeded Tree Species and Their Large-Bodied Avian Frugivores–A Study in Vazhachal Reserve Forest, India. | 2018 | ProQuest Dissertations and Theses: University of Arkansas | Lele, A. A. | wrong location |
| Importance of primary and secondary seed dispersal in the Malagasy tree Commiphora guillaumini | 1999 | Ecology | Böhning-Gaese, K., Gaese, B. H., & Rabemanantsoa, S. B. | no perch |
| Incorporating seed fate into plant-frugivore networks increases interaction diversity across plant regeneration stages | 2016 | Oikos | Donoso, I., García, D., Rodríguez‐Pérez, J., & Martínez, D. | no perch |
| Inducing seed dispersal by generalist frugivores: A new technique to overcome dispersal limitation in restoration | 2020 | Journal of Applied Ecology | Silva, W. R., Zaniratto, C. P., Ferreira, J. O., Rigacci, E. D., Oliveira, J. F., Morandi, M. E., ... & Abreu, L. B. | no control |
| Influence of conspecific and heterospecific adults on riparian tree species establishment during encroachment of a humid palm savanna | 2011 | Oecologia | Rolhauser, A. G., Chaneton, E. J., & Batista, W. B. | wrong location |
| Influence of vegetation structure on spatial patterns of seed deposition by birds | 1999 | New Zealand Journal of Botany | Ferguson, R. N., & Drake, D. R. | no perch |
| Integration of techniques of soil, plants and animals for restoration of degraded areas | 2008 | Ciencia Rural | Regensburger, B., Comin, J. J., & Aumond, J. J. | foreign language |
| Interhabitat variation in diplochory: Seed dispersal effectiveness by birds and ants differs between tropical forest and savanna | 2019 | Perspectives in Plant Ecology, Evolution and Systematics | Camargo, P. H., Rodrigues, S. B., Piratelli, A. J., Oliveira, P. S., & Christianini, A. V. | no perch |
| Internal seed dispersal by parrots: an overview of a neglected mutualism | 2016 | PeerJ | Blanco, G., Bravo, C., Pacifico, E. C., Chamorro, D., Speziale, K. L., Lambertucci, S. A., ... & Tella, J. L. | no perch |
| Intra and inter-annual variation in seed rain in a secondary dry tropical forest excluded from chronic disturbance | 2011 | Forest Ecology and Management | Martínez-Garza, C., Osorio-Beristain, M., Valenzuela-Galván, D., & Nicolás-Medina, A. | no perch |
| Introduction of mammalian seed predators and the loss of an endemic flightless bird impair seed dispersal of the New Zealand tree Elaeocarpus dentatus | 2018 | Ecology and Evolution | Carpenter, J. K., Kelly, D., Moltchanova, E., & O'Donnell, C. F. | no perch |
| Invasion of Rubus praecox (Rosaceae) is promoted by the native tree Aristotelia chilensis (Elaeocarpaceae) due to seed dispersal facilitation | 2015 | *Gayana Botánica* | Rejmánek, M. | no data |
| Isolated pasture trees and the vegetation under their canopies in the Chiapas Coastal Plain, Mexico | 1999 | Biotropica | Otero-Arnaiz, A., Castillo, S., Weave, J., & Ibarra-Manríquez, G. | no control |
| Isolated trees with high crown coverage and densities increase pasture seed rain | 2016 | Acta Botanica Brasilica | Vergne, D. C., Almeida, H. S., Campos, C. C. F., Martins, N., & Ramos, F. N. | no control |
| Iterative increase of economic tree species in managed swidden-fallows of the Amazon | 1990 | Agroforestry Systems | Unruh, J. D. | no perch |
| Landscape reclamation at a central Florida phosphate mine | 1992 | Ecological Engineering | Brown, M. T., Tighe, R. E., McClanahan, T. R., & Wolfe, R. W. | no perch |
| Landscape regeneration by seeds and successional pathways to restore fragile tropandean slopelands | 1997 | Mountain Research and Development | Sarmiento, F O | no control |
| Landscape structure mediates zoochorous-dispersed seed rain under isolated pasture trees across distinct tropical regions | 2019 | Landscape Ecology | Charles, L. S., Dwyer, J. M., Chapman, H. M., Yadok, B. G., & Mayfield, M. M. | no control |
| Land-use and edge effects unbalance seed dispersal and predation interactions under habitat fragmentation | 2011 | Ecological Research | Magrach, A., Guitian, J., Larrinaga, A. R., & Guitián, J. | no perch |
| Limited directed seed dispersal in the canopy as one of the determinants of the low hemiepiphytic figs' recruitments in Bornean rainforests | 2019 | PLoS ONE | Nakabayashi, M., Inoue, Y., Ahmad, A. H., & Izawa, M. | no perch |
| Limited seed dispersal may explain differences in forest colonization by the Japanese raisin tree (Hovenia dulcis thunb.), an invasive alien tree in southern Brazil | 2015 | Tropical Conservation Science | de Sá Dechoum, M., Rejmánek, M., Castellani, T. T., & Zalba, S. M. | no control |
| Linking frugivore activity to early recruitment of a bird dispersed tree, Eugenia umbelliflora (Myrtaceae) in the Atlantic rainforest | 2009 | Austral Ecology | Cortes, M. C., Cazetta, E., Staggemeier, V. G., & Galetti, M. | no data |
| Linking seed dispersal, germination and seedling recruitment in the invasive species Berberis darwinii (Darwin's barberry) | 2008 | PLANT ECOLOGY | McAlpine, K. G., & Jesson, L. K. | no perch |
| Man-made perching sites - electricity pylons accelerate fleshy-fruited plants succession in farmlands | 2017 | Flora: Morphology, Distribution, Functional Ecology of Plants | Dylewski, Łukasz., Kurek, P., Wiatrowska, B., Jerzak, L., & Tryjanowski, P. | no control |
| Monitoring seed dispersal at isolated standing trees in tropical pastures: consequences for local species availability | 1993 | Plant Ecology | Guevara, S., & Laborde, J. | no control |
| Myiarchus flycatchers are the primary seed dispersers of Bursera longipes in a Mexican dry forest | 2016 | PeerJ | Almazán-Nunez, R. C., Eguiarte, L. E., del Coro Arizmendi, M., & Corcuera, P. | no data |
| Negative impact of slash-and-burn agriculture on the seed rain in a tropical dry forest | 2023 | Forest Ecology and Management | Bezerra, J. S., Arroyo-Rodríguez, V., Dupuy-Rada, J. M., Leal, I. R., & Tabarelli, M. | no perch |
| Network models of frugivory and seed dispersal: Challenges and opportunities | 2011 | Acta Oecologica | Carlo, T. A., & Yang, S. | no control |
| Nonredundancy in the dispersal network of a generalist tropical forest tree | 2011 | Ecology | McConkey, K. R., & Brockelman, W. Y. | no perch |
| Nucleation in tropical ecological restoration | 2010 | Scientia Agricola | Reis, A., Bechara, F. C., & Tres, D. R. | no data |
| Nucleation Processes in a Mediterranean Bird-Dispersed Plant | 1996 | Functional Ecology | Verdú, M., & García-Fayos, P. | no control |
| Nucleation-driven regeneration promotes post-fire recovery in a Chilean temperate forest | 2013 | Plant Ecology | Albornoz, F. E., Gaxiola, A., Seaman, B. J., Pugnaire, F. I., & Armesto, J. J. | no data |
| Nurse rocks influence forest expansion over native grassland in southern Brazil | 2011 | Journal of Vegetation Science | Carlucci, M. B., Duarte, L. D. S., & Pillar, V. D. | wrong location |
| Old field vegetation height and the dispersal pattern of bird-disseminated woody plants | 1986 | Bulletin of the Torrey Botanical Club | McDonnell, M. J. | no control |
| Pasture trees contribute to structural heterogeneity and plant distributions in post-agricultural forests decades after canopy closure | 2020 | Journal of Vegetation Science | Holmes, M. A. | no perch |
| Pattern and impact of hornbill seed dispersal at nest trees in a moist evergreen forest in Thailand | 2004 | Journal of Tropical Ecology | Kitamura, S., Yumoto, T., Poonswad, P., Noma, N., Chuailua, P., Plongmai, K., ... & Suckasam, C. | wrong location |
| Patterns of roost site use by Asian hornbills and implications for seed dispersal | 2020 | bioRxiv | Naniwadekar, R., Rathore, A., Shukla, U., & Datta, A. | no control |
| Patterns of seed rain and seedling regeneration in abandoned agricultural clearings in a seasonally dry tropical forest in India | 2010 | Journal of Tropical Ecology | Teegalapalli, K., Hiremath, A. J., & Jathanna, D. | no perch |
| Perfect poopers; passerine birds facilitate sexual reproduction in clonal keystone plants of the boreal forest through directed endozoochory towards dead wood | 2023 | Forest Ecology and Management | Arnberg, M. P., Patten, M. A., Klanderud, K., Haddad, C., Larsen, O., & Steyaert, S. M. | no control |
| Persistence of Seed Dispersal in Agroecosystems: Effects of Landscape Modification and Intensive Soil Management Practices in Avian Frugivores, Frugivory and Seed Deposition in Olive Croplands | 2021 | Frontiers in Ecology and Evolution | Rey, P. J., Camacho, F. M., Tarifa, R., Martínez-Núñez, C., Salido, T., Pérez, A. J., & García, D. | no control |
| Phenology of an understory shrub and bird-dispersal in relation to tree-fall gaps in a subtropical montane forest of Northwest Argentina | 1997 | Ecologia Austral | Pachecho, S., & Grau, R. | foreign language |
| Phenology, seed dispersal and regeneration patterns of Horsfieldia kingii, a rare wild nutmeg | 2013 | Tropical Conservation Science | Datta, A., & Rane, A. | no control |
| Phenology, seed dispersal, and recruitment in cecropia peltata (moraceae) in costa rican tropical dry forest | 1990 | Journal of Tropical Ecology | Fleming, T. H. & Williams, C. F. | no perch |
| Pinon and Juniper Seed Dispersal and Seedling Recruitment at Woodland Ecotones Types of Woodland Ecotones | 1999 | USDA Forest Service Proceedings RMRS-P-11 | Schupp, E. W., Chambers, J. C., Vander Wall, S. B., Gómez, J. M., & Fuentes, M. | no perch |
| Plant-animal relations: effects of disturbance on the regeneration of commercial tree species | 2002 | Tropenbos International | Mbelli, H | no perch |
| Post-Dispersal Seed Predation Rates in a Puerto Rican Pasture | 2013 | Caribbean journal of science | Carlo, T. A., Flores-Mangual, M. L., & Caraballo-Ortiz, M. A. | no control |
| Post-dispersal seed removal in four frugivore-dispersed tree species | 2001 | Monographiae Biologicae Volume 80 | Forget, P. M., Feer, F., Chauvet, S., Julliot, C., Simmen, B., Bayart, F., & Pagès-Feuillade, E. | no perch |
| Pre-dispersal seed predation of the columnar cactus (neobuxbaumia tetetzo, cactaceae) by birds in central Mexico | 2014 | Ornitologia Neotropical | Contreras-González, A. M., & Arizmendi, M. D. C. | no perch |
| Promotion of mid-successional seedling recruitment and establishment by Juniperus virginiana in a coastal environment | 2002 | Plant Ecology | Joy, D A & Young, D R | wrong location |
| Quantitative analysis of seed rain under natural and artificial perches in the Araucaria Forest | 2007 | Brazilian Journal of Forest Research / Pesquisa Florestal Brasileira | Mikich, S. B., & Possette, R. D. S. | foreign language |
| Rain forest expansion mediated by successional processes in vegetation thickets in the Western Ghats of India | 2003 | Journal of Biogeography | Puyravaud, J. P., Dufour, C., & Aravajy, S. | no perch |
| Rain Forest Regeneration beneath the Canopy of Fig Trees Isolated in Pastures of Los Tuxtlas, Mexico | 2004 | Biotropica | Guevara, S., Laborde, J., & Sánchez?Rios, G. | no control |
| Recruitment dynamics of a fleshy-fruited plant (Olea europaea): connecting patterns of seed dispersal to seedling establishment | 2000 | Journal of Ecology | Rey, P. J., & Alcántara, J. M. | no perch |
| Recruitment of two Opuntia species invading abandoned olive groves | 2002 | Acta Oecologica | Gimeno, I., & Vilà, M. | wrong location |
| Rehabilitation of bauxite mines with native forests: the 'traditional method' versus the 'ecological method'. | 2008 | Areas degradadas | Guimarães, J. C. C. | foreign language |
| Restoration demonstrative units using nucleation techniques in Atlantic forest seasonal semidecidual | 2013 | Revista Verde de Agroecologia e Desenvolvimento SustentÃƒÂ¡vel | Alves, M V P & Pinheiro, L B A | foreign language |
| Restoration on abandoned tropical pasturelands - do we know enough? | 2004 | Journal for Nature Conservation | Florentine, S. K., & Westbrooke, M. E. | no data |
| Role of Avian Seed Dispersers in Tree Recruitment in Woodland Pastures | 2017 | Ecosystems | Martínez, D., & García, D. | no perch |
| ROLE OF BIRDS ON THE REGENERATION OF THE WOODY BOSCIA SENEGALENSIS (PERS.) LAM. IN SAHELIAN SAVANNA IN NORTH SENEGAL [Rôle des oiseaux sur la régénération du ligneux Boscia senegalensis (Pers.) Lam. en savane sahélienne au Nord Sénégal in French] | 1997 | Revue d'Écologie (La Terre et La Vie) | Treca, B & Tamba, S | foreign language |
| Roost site use by Great (Buceros bicornis) and Wreathed (Rhyticeros undulatus) Hornbill and its implications for seed dispersal | 2022 | Biotropica | Naniwadekar, R., Rathore, A., Shukla, U., & Datta, A. | no perch |
| Seed and seedling density of Zanthoxylum fagara in Mexico and Zanthoxylum coco in Argentina: influence of distance to vegetation border and plants under which they occur | 2021 | Botanical Sciences | Valdes-Alameda, R., Jurado, E., Flores, J., Pando-Moreno, M., Estrada, E., & Gurvich, D. E. | foreign language |
| Seed and seedling ecology of pinon and juniper species in the pygmy woodlands of western North America | 1999 | The Botanical Review | Chambers, J. C., Vander Wall, S. B., & Schupp, E. W. | no control |
| Seed bank and seed rain of woody species in a Chamaecyparis obtusar dominated suburban secondary forest | 2005 | Journal of the Japanese Society of Revegetation Technology [Nihon Ryokka Kogakkaishi] | Abe, Y., Shibata, S., Nakanishi, A., & Osawa, N. | foreign language |
| Seed deposition by birds on artificial perches at different distances from a gallery forest in the Cerrado area | 2018 | Floresta | de Oliveira, A. K. M., Anghinoni Bocchese, R., Fortes Pereira, K. R., & de Carvalho, T. D. | no control |
| Seed dispersal and vegetation dynamics at a cock-of-the-rock's lek in the tropical forest of French Guiana | 1993 | Journal of Tropical Ecology | Thery, M and Larpin, D | no perch |
| Seed dispersal as an ecosystem service by a keystone avian frugivore in New Zealand | 2020 | New Zealand Journal of Botany | Pegman, A P M | no control |
| Seed Dispersal by Avian Frugivores: Non-random Heterogeneity at Fine Scales | 2015 | Biotropica | Viswanathan, A., Naniwadekar, R., & Datta, A. | no perch |
| Seed dispersal by bats and birds in forest and disturbed habitats of Chiapas, Mexico | 1999 | Biotropica | Medellin, R. A. & Gaona, O. | no perch |
| Seed dispersal by birds and bats in lowland philippine forest successional area | 2009 | Biotropica | Gonzales, R. S., Ingle, N. R., Lagunzad, D. A., & Nakashizuka, T. | no control |
| Seed dispersal by birds and densities of shrubs under trees in arid south Australia | 1987 | Transactions of The Royal Society of South Australia | Tester, M., Paton, D. C., Reid, N., & Lange, R. T. | no control |
| Seed dispersal by macaws shapes the landscape of an Amazonian ecosystem | 2017 | Scientific Reports | Baños-Villalba, A., Blanco, G., Díaz-Luque, J. A., Dénes, F. V., Hiraldo, F., & Tella, J. L. | no perch |
| Seed dispersal by neotropical birds: emerging patterns and underlying processes | 2012 | Ornitologia Neotropical | Karubian, J., Browne, L., Bosque, C., Carlo, T., Galetti, M., Loiselle, B. A., ... & Wikelski, M. | no control |
| Seed dispersal by Salvin's curassow, Mitu salvini (Cracidae), in a tropical forest of Colombia: Direct measurements of dispersal distance | 1999 | Biotropica | Yumoto, T. | no perch |
| Seed dispersal by the Indian grey hornbill Ocyerosbirostris in Eastern Ghats, India | 2011 | Ecotropica | Santhoshkumar, E & Balasubramanian, P | no perch |
| Seed dispersal by the lek-forming white-bearded manakin (Manacus manacus, Pipridae) in the Brazilian Atlantic forest | 2013 | Journal of Tropical Ecology | Cestari, C & Pizo, M A | no perch |
| Seed dispersal by wind, birds, and bats between Philippine montane rainforest and successional vegetation | 2003 | Oecologia | Ingle, N. R. | no perch |
| Seed dispersal limitations shift over time in tropical forest restoration | 2015 | Ecological Applications | Reid, J. L., Holl, K. D., & Zahawi, R. A. | no perch |
| Seed dispersal of a high quality fruit by specialized frugivores: High quality dispersal? | 2000 | Biotropica | Wenny, D G | no perch |
| Seed dispersal of Bursera fagaroides (Burseraceae): The effect of linking environmental factors | 2006 | Southwestern Naturalist | Ortiz-Pulido, R & Rico-Gray, V | no perch |
| Seed dispersal of fleshy-fruited invasive plants by birds: contributing factors and management options | 2005 | Diversity and Distributions | Gosper, C. R., Stansbury, C. D., & Vivian?Smith, G. | no data |
| Seed dispersal of the Chinese tallow tree (Sapium sebiferum (L.) Roxb.) by birds in Coastal South Carolina | 2000 | American Midland Naturalist | Renne, I. J., Gauthreaux Jr, S. A., & Gresham, C. A. | no control |
| Seed dispersal phenology of encroaching woody species in the Free State National Botanical Garden, South Africa | 2022 | African Journal of Ecology | Vukeya, L. R., Mokotjomela, T. M., Malebo, N. J., & Saheed, O. | no control |
| Seed dispersal turns an experimental plantation on degraded land into a novel forest in urban northern Puerto Rico | 2015 | Forest Ecology and Management | Martínez, O. J. A., Ackerman, E. J. M., Montiel, D. G., & Parrotta, J. A. | no perch |
| Seed dispersal, seed predation, and seedling recruitment of a neotropical montane tree | 2000 | Ecological Monographs | Wenny, Daniel G. | no control |
| Seed dissemination by frugivorous birds from forest fragments to adjacent pastures on the western slope of VolcÃ¡n Barva, Costa Rica | 2002 | Revista de Biologia Tropical | Barrantes, G & Pereira, A | no control |
| Seed predation and dispersal in relict Scots pine forests in southern Spain | 1999 | Plant Ecology | Castro, J., Gómez, J. M., García, D., Zamora, R., & Hódar, J. A. | no control |
| Seed rain and seed limitation in a planted gallery forest in Brazil | 2006 | Restoration Ecology | Barbosa, K. C., & Pizo, M. A. | no control |
| Seed rain and seedling establishment of the dioecious tree Neolitsea sericea (Lauraceae): Effects of tree sex and density on invasion into a conifer plantation in central Japan | 2005 | Canadian Journal of Botany | Arai, N & Kamitani, T | no control |
| Seed rain in cocoa agroforests is induced by effects of forest loss on frugivorous birds and management intensity | 2021 | Agriculture, Ecosystems and Environment | Araújo-Santos, I., Morante-Filho, J. C., Oliveira, S., Cabral, J. P., Rocha-Santos, L., Cassano, C. R., ... & Benchimol, M. | no perch |
| Seed rain of fleshy and dry propagules in different habitats in the temperate rainforests of Chiloé Island, Chile | 2001 | Austral Ecology | Armesto, J. J., Díaz, I., Papic, C., & Willson, M. F. | no perch |
| Seed rain of fleshy-fruited species in tropical pastures in Los Tuxtlas, Mexico | 2002 | Journal of Tropical Ecology | Martínez-Garza, C., & González-Montagut, R. | no perch |
| Seed rain produced by bats and birds in forest islands in a Neotropical Savanna | 2006 | Biotropica | Arteaga, L. L., Aguirre, L. F., & Moya, M. I. | no perch |
| Seed transfer among bird-dispersed trees and its consequences for post-dispersal seed fate | 2007 | Basic and Applied Ecology | García, D., Martínez, I., & Obeso, J. R. | no control |
| Seed-fall in the early stage of succession after a forest fire | 1988 | Ecological Review | Kominami, Y | no perch |
| Seedling establishment after prescribed burning of a clear-cut and a partially cut mesic boreal forest in southern Finland | 1996 | Silva Fennica | Vanha-Majamaa, I., Tuittila, E., Tonteri, T., & Suominen, R. | no perch |
| Seedling recruitment under isolated trees in a tea plantation provides a template for forest restoration in eastern Africa | 2021 | PLoS ONE | Ndangalasi, H. J., Martínez-Garza, C., Harjo, T. C., Pedigo, C. A., Wilson, R. J., & Cordeiro, N. J. | no control |
| Seeing the forest through the weeds: frugivorous birds and rainforest regeneration in subtropical regrowth dominated by camphor laurel | 2006 | 15th Australian Weeds Conference Papers and Proceedings | Neilan, W., Catterall, C., Kanowski, J., & McKenna, S. | no perch |
| Selective clearing: forest regeneration through an ancestral agricultural practice. | 2002 | Bois et Forêts des Tropiques | Carrière, S. M. | foreign language |
| Shrub vs. grass-patch effects on the seed rain and seed bank of a five-year pasture in Puerto Rico | 2006 | Ecotropica | Myster, R. W. | no bird |
| Spanish juniper gain expansion opportunities by counting on a functionally diverse dispersal assemblage community | 2013 | Ecology and Evolution | Escribano‐Ávila, G., Pías, B., Sanz‐Pérez, V., Virgós, E., Escudero, A., & Valladares, F. | no perch |
| Spatial analyses of invasion patterns of Chinese Tallow (Triadica sebifera) in a Wet Slash Pine (Pinus elliottii) flatwood in the coastal plain of Mississippi, USA | 2018 | Forest Science | Fan, Z. | no perch |
| Spatial and seasonal patterns of seed dissemination of Cornus controversa in a temperate forest | 1994 | Ecology | Masaki, T., Kominami, Y., & Nakashizuka, T. | no control |
| Spatial and temporal effects on recruitment of an Afromontane forest tree in a threatened fragmented ecosystem | 2009 | Biological Conservation | Lehouck, V., Spanhove, T., Gonsamo, A., Cordeiro, N., & Lens, L. | no perch |
| Spatial and temporal variation of seed rain in the canopy and on the ground of a tropical cloud forest | 2013 | Biotropica | Sheldon, K S & Nadkarni, N M | no perch |
| Spatial concordance between seed rain and seedling establishment in bird-dispersed trees: Does scale matter? | 2005 | Journal of Ecology | Garcia, D., Obeso, J. R., & Martínez, I. | no control |
| Spatial distribution patterns of Rhus coriaria seedlings after fire in a Mediterranean pine forest | 1992 | Acta Oecologica | Izhaki, I., Lahav, H., & Ne'Eman, G. | missing |
| Spatial heterogeneity of a parasitic plant drives the seed-dispersal pattern of a zoochorous plant community in a generalist dispersal system | 2016 | Functional Ecology | Mellado, A & Zamora, R | wrong location |
| Spatial networks of fleshy-fruited trees drive the flow of avian seed dispersal through a landscape | 2014 | Functional Ecology | Rodríguez‐Pérez, J., García, D., & Martínez, D. | no control |
| Spatial pattern of bird-dispersed seed rain ofdaphniphyllum macropodum in an evergreen broad-leaved forest | 1997 | Journal of Sustainable Forestry | Kominami, Y., Tanouchi, H., & Sato, T. | no perch |
| Spatial pattern of seed rain of fleshy-fruited plants in a scrubland-grassland transition | 1995 | Acta Oecologica | Kollmann, J & Pirl, M | no perch |
| Spatial-temporal distribution of ornithochorous seeds from an Elaeagnus umbellata community dominating a riparian habitat | 2011 | Plant Species Biology | Kohri, M., Kamada, M., & Nakagoshi, N. | no control |
| Spatiotemporal variation of a Pinus seed rain available for an endemic finch in an insular environment | 2011 | European Journal of Wildlife Research | Garcia-del-Rey, E., Nanos, N., López-de-Heredia, U., Muñoz, P. G., Otto, R., Fernández-Palacios, J. M., & Gil, L. | no perch |
| Stimulating natural regeneration of tropical forest on degraded land: Approaches, outcomes, and information gaps | 2013 | Restoration Ecology | Shoo, L. P., & Catterall, C. P. | no data |
| Strategies for the recovery of degraded ecosystems: experiences from Latin America | 2001 | Interciencia | Montagnini, F | no perch |
| Subannual phenology and the effect of staggered fruit ripening on dispersal competition | 2022 | Biotropica | Schubert, S C & Walters, E L | no control |
| Temporal and spatial patterns of woody plant establishment in Michigan old fields | 1999 | American Midland Naturalist | Foster, B L & Gross, K L | no control |
| Testing applied nucleation as a strategy to facilitate tropical forest recovery | 2013 | Journal of Applied Ecology | Zahawi, R. A., Holl, K. D., Cole, R. J., & Reid, J. L. | no control |
| The biology of fig trees and their associated animals | 1996 | Journal of Biogeography | Compton, S. G., Wiebes, J. T., & Berg, C. C. | no perch |
| The contribution of jays to oak regeneration in a naturalistically managed forest in the Lünburg Heath | 1995 | Forst und Holz | Vullmer, H & Hanstein, U | foreign language |
| The diversity and conservation of plant reproductive and dispersal functional traits in human-dominated tropical landscapes | 2006 | Journal of Ecology | Mayfield, M. M., Ackerly, D., & Daily, G. C. | no perch |
| The effects of microsite (logs versus ground surface) on the presence of forest floor biota in a second-growth hardwood forest | 2002 | General Technical Report - Pacific Southwest Research Station, USDA Forest Service | Pyle, C & Brown, M M | no data |
| The Hornbill (Tockus semifasciatus) as a seed-disperser and ecological indicator, and forest rehabilitation in eastern Ivory Coast. | 1999 | Revue d'Écologie (La Terre et La Vie) | Jensch, D & Ellenberg, H | foreign language |
| The Importance of Ficus (Moraceae) Trees for Tropical Forest Restoration | 2016 | Biotropica | Cottee?Jones, H. E. W., Bajpai, O., Chaudhary, L. B., & Whittaker, R. J. | no control |
| The importance of seed dispersal in the Alexandria Coastal Dunefield, South Africa | 2001 | Journal of Coastal Conservation | Castley, J. G., Bruton, J. S., Kerley, G. I. H., & McLachlan, A. | wrong location |
| The role of animal seed dispersal in accelerating native forest regeneration on degraded tropical lands | 1997 | Forest Ecology and Management | Wunderle, J M | no data |
| The role of arboreal Seed dispersal groups on the Seed rain of a lowland tropical forest | 2001 | Biotropica | Clark, C J, Poulsen, J R & Parker, V T | no perch |
| The role of birds in seed dispersal and its consequences for forest ecosystems | 2006 | (conference proceeding) | Bleher, B. & Bhning-Gaese, K. | no perch |
| The role of frugivorous birds and bats in the colonization of cloud forest plant species in burned areas in western Mexico | 2015 | Animal Biodiversity and Conservation | Rost, J., Jardel-Peláez, E. J., Bas, J. M., Pons, P., Loera, J., Vargas-Jaramillo, S., & Santana, E. | no perch |
| The Role of Isolated Trees in Facilitating Tree Seedling Recruitment at a Degraded Sub‐Tropical Rainforest Site | 1999 | Restoration ecology | Toh, I., Gillespie, M., & Lamb, D. | no data |
| The role of remnant trees in seed dispersal through the matrix: Being alone is not always so sad | 2009 | Biological Conservation | Herrera J & García D | no control |
| The role of seed dispersal, seed predation and drought in the restoration of Ngel Nyaki Forest, Nigeria. | 2014 | School of Biological Sciences, University of Canterbury, Christchurch, New Zealand | Roselli, S. M. | no data |
| The use of grandiúva, Trema micrantha Blume (Ulmaceae), in the restoration of degraded areas: the role of birds that eat its fruits [O uso da grandiuva, Trema micrantha Blume (Ulmaceae), na recuperacao de areas degradadas: o papel das aves que se alimentam de seus frutos] | 2001 | Tangara | Zimmermann, C E | foreign language |
| The use of nucleation techniques to restore the environment: a bibliometric analysis | 2014 | Natureza & Conservação | Boanares, D., & de Azevedo, C. S. | no data |
| Traits of perch trees promote seed dispersal of endemic fleshy-fruit species in degraded areas of endangered Mediterranean ecosystems | 2019 | Journal of Arid Environments | Miranda, A., Vásquez, I. A., Becerra, P., Smith-Ramírez, C., Delpiano, C. A., Hernández-Moreno, A., & Altamirano, A. | no control |
| Tree and shrub seed dispersal in pastures: The importance of rainforest trees outside forest fragments | 2008 | Ecoscience | Laborde, J., Guevara, S., & Sánchez-Ríos, G. | no control |
| Tree canopies facilitate invasion of communal savanna rangelands by Lantana camara | 2002 | African Journal of Range & Forage Science | Rodger, J G & Twine, W C | no perch |
| Trophic interactions and feedbacks maintain intact and degraded states of Hawaiian tropical forests | 2022 | Ecosphere | Yelenik, S, Rose, E, & Paxton, E H | no perch |
| Tropical forests: structure, diversity and function - part B. Restoration ecology | 2001 | Tropical ecosystems: structure, diversity and human welfare. Proceedings of the International Conference on Tropical Ecosystems: Structure, Diversity and Human Welfare, Banglore, India, 15-18 July, 2001 | Ganeshaiah, K. N., Shaanker, R. U., & Bawa, K. S. | no perch |
| Tropical wet forest treefall gaps and distributions of understory birds and plants | 1988 | Ecology | Levey, D J | no perch |
| Uncovering mechanisms of bird seed dispersal in semiarid environments to help to restore them | 2019 | Ecosphere | Martínez‐López, V., Zapata, V., De la Rúa, P., & Robledano, F. | no control |
| nonredundancy in the dispersal network of a generalist tropical forest tree | 2018 | Neotropical Biology and Conservation | Vogel, H. F., McCarron, V. E. A., & Zocche, J. J. | no data |
| Variation in mistletoe seed deposition: effects of intra- and interspecific host characteristics | 2002 | Ecography | Aukema, J. E., & Del Rio, C. M. | no control |
| Variation in woody plant species establishment according to nurse plant size in the South African grassland | 2016 | Taiwania | Fujita, T., & Yamashina, C. | wrong location |
| Where do seeds go when they go far? Distance and directionality of avian seed dispersal in heterogeneous landscapes | 2013 | Ecology | Carlo, T. A., García, D., Martínez, D., Gleditsch, J. M., & Morales, J. M. | no perch |
| Where does a fruit-eating bird deposit mistletoe seeds? Seed deposition patterns and an experiment | 2002 | Ecology | Aukema, J. E. & del Rio, C. M. | no perch |
| Windbreaks enhance seed dispersal into agricultural landscapes in Monteverde, Costa Rica. | 2000 | Ecological Applications | Harvey, C. A. | no control |

**Table 3. List of excluded articles after critical appraisal.**

| **Study** | **Reason** | **Details** |
| --- | --- | --- |
| Knight, R. S. (1988). Aspects of plant dispersal in the southwestern Cape with particular reference to the roles of birds as dispersal agents. | invalid control | Control doesn't qualify as seed deposition is excluded. No measure of variance. |
| Martínez, I., García, D., & Obeso, J. R. (2008). Differential seed dispersal patterns generated by a common assemblage of vertebrate frugivores in three fleshy-fruited trees. Ecoscience, 15(2), 189-199. | invalid location | focus was on the dispersal of selected species by looking at different microhabitat sites in the landscape, including covered and uncovered ones. Mean seed densities were unclear for the grouped microsites; the perch effect is unclear because the trees are inside the forest fragments |
| Giertych, P. (2000). Factors determining natural regeneration of yew (Taxus baccata L.) in the Kórnik Arboretum. Dendrobiology, 45. | distributional data | did not collect data specifically for perch / microhabitat; looked at plant distribution and their habitat |
| Carlo, T. A., & Morales, J. M. (2016). Generalist birds promote tropical forest regeneration and increase plant diversity via rare‐biased seed dispersal. Ecology, 97(7), 1819-1831. | invalid control | did not have proper control; 'control' sites excluded bird seed rain by putting nets over experimental patches |
| Deckers, B., Verheyen, K., Vanhellemont, M., Maddens, E., Muys, B., & Hermy, M. (2008). Impact of avian frugivores on dispersal and recruitment of the invasive Prunus serotina in an agricultural landscape. Biological Invasions, 10, 717-727. | invalid control | no control; reported hedgerow site |
| Piña-Rodrigues, F. C., Piratelli, A. J., Rudge, A. C., Gondim, F., Freire, M., & Correa, J. (2009). Mobile links in fragmented ecosystem: seed and birds dispersal approach towards Atlantic forest restoration and conservation. Biodiversity and land use systems in the fragmented Mata Atlântica of Rio de Janeiro. Göttingen: Cuvillier Verlag, 313-360. | sample size | no replicates for the treatment; compared one natural perch with no perches |
| Nishi, H., & Tsuyuzaki, S. (2004). Seed dispersal and seedling establishment of Rhus trichocarpa promoted by a crow (Corvus macrorhynchos) on a volcano in Japan. Ecography, 27(3), 311-322. | invalid control | used only 1 belt transect as method |
| Vasconcellos-Neto, J., Albuquerque, L. B. D., & Silva, W. R. (2009). Seed dispersal of Solanum thomasiifolium Sendtner (Solanaceae) in the Linhares Forest, Espírito Santo state, Brazil. Acta Botanica Brasilica, 23, 1171-1179. | invalid outcome | wrong outcome; presented percentage of seed in the faeces |
| Jordano, P., & Schupp, E. W. (2000). Seed disperser effectiveness: the quantity component and patterns of seed rain for Prunus mahaleb. Ecological monographs, 70(4), 591-615. | invalid location | not degraded area (no matrix); looked at microhabitat sites within the reserve |
| Puig, H., Fabre, A., & Gauquelin, T. (1998). Spatial distribution of seedlings and young plants of Iryanthera hostmannii (Benth.) Warb. in French Guyana tropical rain forest. Comptes Rendus de l'Academie des Sciences Series III Sciences de la Vie, 5(321), 429-435. | foreign language | data in foreign language section |
| Calviño-Cancela, M. (2002). Spatial patterns of seed dispersal and seedling recruitment in Corema album (Empetraceae): the importance of unspecialized dispersers for regeneration. Journal of Ecology, 775-784. | distributional data | did not collect data specifically for perch / microhabitat, but noted the microhabitat for each bird faeces; outcome also not certain as the study estimated no. of seeds by multiplying no. of faeces with average seed contents |
| Graham, L. L. B., Salahunddin, Limin, S., & Page, S. (2006). The role of artificial bird perches for increasing seed dispersal in degraded tropical peat swamp forest. Funding: Rufford Small Grant for Nature Conservation, 16–27. | redundant data | data report only; use the published article instead, which was also included in the accepted studies (Graham, 2012) |
| Thomas, D. W., Cloutier, D., Provencher, M., & Houle, C. (1988). The shape of bird-and bat-generated seed shadows around a tropical fruiting tree. Biotropica, 20(4), 347-348. | sample size | no replicate for the intervention; just looked at one tree |
| González‐Varo, J. P., Carvalho, C. S., Arroyo, J. M., & Jordano, P. (2017). Unravelling seed dispersal through fragmented landscapes: Frugivore species operate unevenly as mobile links. Molecular Ecology, 26(16), 4309-4321. | invalid control | used a single transect for control, no replicate |
| Torre, R. D., Jiménez, M. D., Ramírez, Á., Mola, I., Casado, M. A., & Balaguer, L. (2015). Use of restoration plantings to enchance bird seed dispersal at the roadside: failures and prospects. Journal of Environmental Engineering and Landscape Management, 23(4), 302-311. | invalid outcome | wrong outcome; no seed data available, only bird droppings (where only 9% have seeds) |
| La Mantia, T., Rühl, J., Massa, B., Pipitone, S., Lo Verde, G., & Bueno, R. S. (2019). Vertebrate‐mediated seed rain and artificial perches contribute to overcome seed dispersal limitation in a Mediterranean old field. Restoration Ecology, 27(6), 1393-1400. | invalid control | no control; ground traps received rabbit & rat dispersed seeds |
| de Sá Dechoum, M., Rejmánek, M., Castellani, T. T., & Zalba, S. M. (2015). Limited seed dispersal may explain differences in forest colonization by the Japanese raisin tree (Hovenia dulcis Thunb.), an invasive alien tree in Southern Brazil. Tropical Conservation Science, 8(3), 610-622. | invalid perch | wrong study design; plot level comparison of treatments |
| Blix, L., Fritioff, Å., & Rinman, U. (2001). The role of remnant trees for patterns of seed dispersal in tropical rain forest in Samoa. Swedish University of Agricultural Sciences. | redundant data | same data presented in another included paper by Elmqvist (2002) |
| Milton, M. (2007). Breaking Barriers of Regeneration: Examining the Effectiveness of Bird Perches in Facilitating Seed Dispersal in a Tropical Dry Forest. Theses. University of Missouri - St. Louis. | no data | States that total seeds were higher where there was a perch but did not report any relevant data to support this. |
| Willson, M. F., & Crome, F. H. J. (1989). Patterns of seed rain at the edge of a tropical Queensland rain forest. Journal of Tropical Ecology, 5(3), 301-308. | no control | No data for control and no variances provided |
| Ramaswami, G., & Sukumar, R. (2011). Woody plant seedling distribution under invasive Lantana camara thickets in a dry-forest plot in Mudumalai, southern India. Journal of Tropical Ecology, 27(4), 365-373. | invalid location | matrix is unclear; they compared seed rain in areas with and without L.camara thickets (potential natural perch), but both treatments are assumingly inside the forest |
| García, D. (2001). Effects of seed dispersal on Juniperus communis recruitment on a Mediterranean mountain. Journal of Vegetation Science, 12(6), 839-848. | distributional data | did not collect data useful for perch vs no perch comparison, but noted the seed rain for certain microhabitats (very small; < 1 m to several m in diameter) |
| García, C., Jordano, P., Arroyo, J. M., & Godoy, J. A. (2009). Maternal genetic correlations in the seed rain: effects of frugivore activity in heterogeneous landscapes. Journal of Ecology, 97(6), 1424-1435. | distributional data | did not collect data useful for perch vs no perch comparison, but noted the seed rain for certain microhabitats (defined based on the cover above 0.16m2 traps) that are close to each other |
