# Supplementary material for: Effectiveness of perches in promoting bird-mediated seed dispersal for natural forest regeneration: a systematic review
Source: Environ Evid. 2025 Jun 14;14:10. doi: 10.1186/s13750-025-00363-8 (PMC12166613; doi:10.1186/s13750-025-00363-8)
Supplement: Supplementary file 4 — Supplementary Material 4: Additional File 4: AF4_meta-analysis_supplementary. The supporting documents for the meta-analysis, including results of meta-regression analysis and sensitivity analysis. [file 13750_2025_363_MOESM4_ESM.docx]

**Effectiveness of perches in promoting bird-mediated seed dispersal for natural forest regeneration: A systematic review**

**Additional File 4.** The supporting documents for the meta-analysis, including results of meta-regression analysis and sensitivity analysis. This file provides supplementary analysis on the comparison of different model structures, subgroup analysis according to risk of bias and method validity, influence of outliers, and publication bias tests. It contains the following:

Table 1. Results of the LRT comparison between three-level models and models with constrained variances for the four outcomes

Table 2. Results of the three-level model for the four outcomes without any moderators

Figure 1. Summary of risk of bias assessment per criteria for each of the four outcomes.

Table 3. Comparison of meta-analysis results using subgroups according to risk of bias for the four outcomes using models with no moderators

Table 4. Comparison of meta-analysis results using subgroups according to risk of bias scores for the four outcomes, with perch type as moderator in the model

Table 5. Comparison of meta-analysis results using subgroups according to method validity for the four outcomes using models with no moderators

Table 6. Comparison of meta-analysis results using subgroups according to method validity for the four outcomes, with perch type as moderator in the model

Table 7. Results of the three-level model for the four outcomes with matrix type as moderator

Table 8. Comparison of meta-analysis results using subgroups according to risk of bias scores for the four outcomes, with matrix type as moderator in the model

Table 9. Comparison of meta-analysis results using subgroups according to risk of bias scores (RoB) for the four outcomes, with biome as moderator in the model

Table 10. Results of the three-level model for the seedling outcomes with precipitation category as moderator.

Table 11. Results of the multilevel meta-regression analysis with perch type, square root of the inverse of effective sample size (sqrt_inv_n_tilda) and publication year as the moderators for the four outcomes tested.

Table 12. Results of the multilevel meta-regression analysis without modifiers using dataset with and without outliers.

Table 13. Results of the multilevel meta-regression analysis according to perch type using dataset with and without outliers.

Table 14. Comparison of the multilevel meta-regression analysis for seedling density with and without variance imputation

Figure 2. Scatterplot of the effect estimates on seed richness over publication year.

Figure 3. Funnel plots of meta-analytic fixed effects models without moderators for each of the four outcomes

Table 1. Results of the LRT comparison between three-level models and models with constrained variances for the four outcomes. Fit and summary statistics Akaike information criterion (AIC), Bayesian information criterion (BIC), log likelihood (LogLik), likelihood ratio test statistics (LRT), and the p-value are provided.

|  | df | AIC | BIC | LogLik | LRT | p-value |
| --- | --- | --- | --- | --- | --- | --- |
| Seed density |  |  |  |  |  |  |
| Three-level model | 3 | 438.87 | 448.08 | -216.43 | NA | NA |
| Within-studies variance constrained | 2 | 454.29 | 460.43 | -225.14 | 17.42 | 0.00 |
| Between-studies variance constrained | 2 | 498.44 | 504.58 | -247.22 | 61.57 | 0.00 |
| Both variance components constrained | 1 | 1320.71 | 1323.78 | -659.36 | 885.84 | 0.00 |
|  |  |  |  |  |  |  |
| Seed richness |  |  |  |  |  |  |
| Three-level model | 3 | 184.71 | 190.56 | -89.35 | NA | NA |
| Within-studies variance constrained | 2 | 188.20 | 192.10 | -92.10 | 5.49 | 0.02 |
| Between-studies variance constrained | 2 | 214.98 | 218.88 | -105.49 | 32.27 | 0.00 |
| Both variance components constrained | 1 | 554.83 | 556.78 | -276.42 | 374.13 | 0.00 |
|  |  |  |  |  |  |  |
| Seedling density |  |  |  |  |  |  |
| Three-level model | 3 | 252.25 | 259.68 | -123.13 | NA | NA |
| Within-studies variance constrained | 2 | 401.53 | 406.49 | -198.77 | 151.28 | 0.00 |
| Between-studies variance constrained | 2 | 269.38 | 274.33 | -132.69 | 19.12 | 0.00 |
| Both variance components constrained | 1 | 565.57 | 568.05 | -281.78 | 317.32 | 0.00 |
|  |  |  |  |  |  |  |
| Seedling richness |  |  |  |  |  |  |
| Three-level model | 3 | 82.48 | 86.68 | -38.24 | NA | NA |
| Within-studies variance constrained | 2 | 103.41 | 106.21 | -49.70 | 22.93 | 0.00 |
| Between-studies variance constrained | 2 | 86.35 | 89.15 | -41.18 | 5.88 | 0.02 |
| Both variance components constrained | 1 | 134.90 | 136.31 | -66.45 | 56.43 | 0.00 |

Table 2. Results of the three-level model for the four outcomes without any moderators. Estimates are presented as standardized mean differences Hedges’ g with their corresponding upper (UB) and lower (LB) 95% confidence intervals, sample size (k), and measure of total heterogeneity I^2^ (I^2^_total).

| **Outcome** | **k** | **g** | **LB** | **UB** | **I^2^_total** |
| --- | --- | --- | --- | --- | --- |
| seed density | 160 | 1.26 | 1.02 | 1.50 | 90.51 |
| seed richness | 53 | 2.16 | 1.47 | 2.84 | 94.28 |
| seedling density | 89 | 1.09 | 0.75 | 1.44 | 90.85 |
| seedling richness | 31 | 0.80 | 0.36 | 1.25 | 83.35 |

Figure 1. Summary of risk of bias assessment per criteria for each of the four outcomes.

Table 3. Comparison of meta-analysis results using subgroups according to risk of bias for the four outcomes using models with no moderators. Estimates are presented as standardized mean differences Hedges’ g with their corresponding upper (UB) and lower (LB) 95% confidence intervals. Summary statistics for the meta-regression and the Cochran’s test for heterogeneity Q are also provided. Highlight in peach indicate changes in the significance of the effect across subgroups.

| seed density | | | | | | | | | | | |
| --- | --- | --- | --- | --- | --- | --- | --- | --- | --- | --- | --- |
|  | g | V | SE | LB | UB | Z | pval | Q | df | Q pval. | rho |
| medium_RoB | 1.1759 | 0.0696 | 0.2638 | 0.6588 | 1.693 | 4.4571 | 0 | 303.2932 | 70 | 0 | 0 |
| high_RoB | 1.2977 | 0.0158 | 0.1258 | 1.0512 | 1.5442 | 10.3181 | 0 | 927.637 | 88 | 0 | 0 |
| All | 1.2578 | 0.0149 | 0.1219 | 1.0188 | 1.4968 | 10.3145 | 0 | 1316.07 | 159 | 0 | 0 |
|  |  |  |  |  |  |  |  |  |  |  |  |
| seed richness | | | | | | | | | | | |
|  | estimate | V | SE | CI.LB | CI.UB | Z | pval | Q | df | Q pval. | rho |
| medium_RoB | 1.6189 | 0.1239 | 0.352 | 0.929 | 2.3088 | 4.5991 | 0 | 278.0891 | 35 | 0 | 0 |
| high_RoB | 3.6446 | 1.1842 | 1.0882 | 1.5117 | 5.7775 | 3.3491 | 8e-04 | 156.9314 | 16 | 0 | 0 |
| All | 2.1601 | 0.1223 | 0.3497 | 1.4747 | 2.8455 | 6.1769 | 0 | 529.5888 | 52 | 0 | 0 |
|  |  |  |  |  |  |  |  |  |  |  |  |
| seedling density | | | | | | | | | | | |
|  | estimate | V | SE | CI.LB | CI.UB | Z | pval | Q | df | Q pval. | rho |
| medium_RoB | 1.2696 | 0.0935 | 0.3058 | 0.6702 | 1.869 | 4.1513 | 0 | 96.443 | 20 | 0 | 0 |
| high_RoB | 1.0066 | 0.0482 | 0.2196 | 0.5762 | 1.4369 | 4.5841 | 0 | 444.9123 | 67 | 0 | 0 |
| All | 1.0912 | 0.0309 | 0.1758 | 0.7465 | 1.4358 | 6.2053 | 0 | 567.2707 | 88 | 0 | 0 |
|  |  |  |  |  |  |  |  |  |  |  |  |
| seedling richness | | | | | | | | | | | |
|  | estimate | V | SE | CI.LB | CI.UB | Z | pval | Q | df | Q pval. | rho |
| medium_RoB | 0.7089 | 0.0399 | 0.1998 | 0.3172 | 1.1006 | 3.5474 | 4e-04 | 75.3784 | 12 | 0 | 0 |
| high_RoB | 0.8402 | 0.2045 | 0.4522 | -0.0462 | 1.7265 | 1.8579 | 0.0632 | 46.3303 | 17 | 2e-04 | 0 |
| All | 0.8045 | 0.0513 | 0.2265 | 0.3606 | 1.2484 | 3.5522 | 4e-04 | 124.7782 | 30 | 0 | 0 |

Table 4. Comparison of meta-analysis results using subgroups according to risk of bias scores for the four outcomes, with perch type as moderator in the model. Estimates are presented as standardized mean differences Hedges’ g with their corresponding upper (UB) and lower (LB) 95% confidence intervals and other test statistics. Highlighted in green are significant effects while in orange are non-significant (p<0.05).

| outcome | perch type | ROB | k | g | se | zval | pval | LB | UB |
| --- | --- | --- | --- | --- | --- | --- | --- | --- | --- |
| seed density | artificial | medium | 13 | 0.98 | 0.68 | 1.44 | 0.15 | -0.35 | 2.31 |
|  |  | high | 15 | 1.29 | 0.22 | 5.83 | 0.00 | 0.85 | 1.72 |
|  | natural | medium | 47 | 1.29 | 0.35 | 3.7 | 0.00 | 0.61 | 1.97 |
|  |  | high | 69 | 1.25 | 0.15 | 8.16 | 0.00 | 0.95 | 1.55 |
|  | semi-natural | medium | 11 | 0.96 | 0.67 | 1.42 | 0.15 | -0.36 | 2.28 |
|  |  | high | 5 | 1.74 | 0.42 | 4.19 | 0.00 | 0.93 | 2.56 |
| seed richness | artificial | medium | 6 | 2.21 | 0.79 | 2.80 | 0.01 | 0.66 | 3.75 |
|  |  | high | 6 | 2.64 | 0.68 | 3.88 | 0.00 | 1.31 | 3.98 |
|  | natural | medium | 26 | 1.47 | 0.49 | 3.00 | 0.00 | 0.51 | 2.43 |
|  |  | high | 10 | 2.65 | 0.67 | 3.97 | 0.00 | 1.34 | 3.96 |
|  | semi-natural | medium | 4 | 1.29 | 1.09 | 1.18 | 0.24 | -0.85 | 3.42 |
|  |  | high | 1 | 13.59 | 2.34 | 5.81 | 0.00 | 9.00 | 18.17 |
| seedling density | artificial | medium | 5 | 1.67 | 0.7 | 2.38 | 0.02 | 0.3 | 3.04 |
|  |  | high | 8 | 0.24 | 0.54 | 0.46 | 0.65 | -0.81 | 1.3 |
|  | natural | medium | 10 | 1.19 | 0.45 | 2.65 | 0.01 | 0.31 | 2.07 |
|  |  | high | 56 | 1.21 | 0.25 | 4.78 | 0.00 | 0.71 | 1.71 |
|  | semi-natural | medium | 6 | 1.05 | 0.7 | 1.5 | 0.13 | -0.32 | 2.43 |
|  |  | high | 4 | 0.56 | 0.58 | 0.96 | 0.34 | -0.58 | 1.69 |
| seedling richness | artificial | medium | 3 | 0.49 | 0.39 | 1.25 | 0.21 | -0.28 | 1.25 |
|  |  | high | 4 | 0.54 | 1.16 | 0.47 | 0.64 | -1.73 | 2.81 |
|  | natural | medium | 10 | 0.79 | 0.24 | 3.31 | 0.00 | 0.32 | 1.26 |
|  |  | high | 14 | 0.94 | 0.59 | 1.58 | 0.11 | -0.23 | 2.10 |

Table 5. Comparison of meta-analysis results using subgroups according to method validity for the four outcomes using models with no moderators. Estimates are presented as standardized mean differences Hedges’ g with their corresponding upper (UB) and lower (LB) 95% confidence intervals. Summary statistics for the meta-regression and the Cochran’s test for heterogeneity Q are also provided.

| seed density | | | | | | | | | | | |
| --- | --- | --- | --- | --- | --- | --- | --- | --- | --- | --- | --- |
|  | estimate | V | SE | LB | UB | Z | pval | Q | df | Q pval | rho |
| low_val | 1.1989 | 0.0337 | 0.1836 | 0.8391 | 1.5588 | 6.5299 | 0 | 199.7596 | 41 | 0 | 0 |
| medium_val | 1.2198 | 0.0188 | 0.1372 | 0.9509 | 1.4887 | 8.891 | 0 | 231.3142 | 57 | 0 | 0 |
| high_val | 1.6769 | 0.208 | 0.4561 | 0.7829 | 2.5709 | 3.6765 | 2e-04 | 466.2286 | 59 | 0 | 0 |
|  |  |  |  |  |  |  |  |  |  |  |  |
| seed richness | | | | | | | | | | | |
|  | estimate | V | SE | LB | UB | Z | pval | Q | df | Q pval | rho |
| low_val | 1.8634 | 0.0959 | 0.3096 | 1.2565 | 2.4703 | 6.0177 | 0 | 84.7178 | 30 | 0 | 0 |
| medium_val | 2.6676 | 0.6183 | 0.7863 | 1.1264 | 4.2088 | 3.3925 | 7e-04 | 369.1931 | 21 | 0 | 0 |
| high_val | NA | NA | NA | NA | NA | NA | NA | NA | NA | NA | NA |
|  |  |  |  |  |  |  |  |  |  |  |  |
| seedling density | | | | | | | | | | | |
|  | estimate | V | SE | LB | UB | Z | pval | Q | df | Q pval | rho |
| low_val | 1.0201 | 0.0637 | 0.2524 | 0.5254 | 1.5149 | 4.041 | 1e-04 | 284.2619 | 43 | 0 | 0 |
| medium_val | 1.3881 | 0.1691 | 0.4112 | 0.5823 | 2.194 | 3.376 | 7e-04 | 176.6043 | 17 | 0 | 0 |
| high_val | 0.9084 | 0.0737 | 0.2715 | 0.3762 | 1.4406 | 3.3456 | 8e-04 | 61.516 | 26 | 1e-04 | 0 |
|  |  |  |  |  |  |  |  |  |  |  |  |
| seedling richness | | | | | | | | | | | |
|  | estimate | V | SE | LB | UB | Z | pval | Q | df | Q pval | rho |
| low_val | 0.3389 | 0.0233 | 0.1526 | 0.0398 | 0.638 | 2.2207 | 0.0264 | 13.3237 | 9 | 0.1485 | 0 |
| medium_val | 0.6034 | 0.0324 | 0.1799 | 0.2508 | 0.956 | 3.3538 | 8e-04 | 50.2038 | 11 | 0 | 0 |
| high_val | 1.7329 | 0.463 | 0.6804 | 0.3993 | 3.0666 | 2.5467 | 0.0109 | 29.0446 | 8 | 3e-04 | 0 |

Table 6. Comparison of meta-analysis results using subgroups according to method validity for the four outcomes, with perch type as moderator in the model. Estimates are presented as standardized mean differences Hedges’ g with their corresponding upper (UB) and lower (LB) 95% confidence intervals and other test statistics. Highlighted in green are significant effects while in orange are non-significant (p<0.05).

| **outcome** | **perch type** |  | **met_val** | **k** | **g** | **se** | **zval** | **pval** | **LB** | **UB** |
| --- | --- | --- | --- | --- | --- | --- | --- | --- | --- | --- |
| seed density | artificial |  | low | 6 | 1.35 | 0.32 | 4.27 | 0 | 0.73 | 1.97 |
|  |  |  | medium | 21 | 1.24 | 0.22 | 5.6 | 0 | 0.81 | 1.67 |
|  |  |  | high | 1 | 1.15 | 2.03 | 0.56 | 0.57 | -2.84 | 5.13 |
|  | natural |  | low | 30 | 1.22 | 0.22 | 5.51 | 0 | 0.79 | 1.65 |
|  |  |  | medium | 35 | 1.17 | 0.18 | 6.6 | 0 | 0.82 | 1.52 |
|  |  |  | high | 51 | 1.83 | 0.62 | 2.96 | 0 | 0.62 | 3.03 |
|  | semi-natural |  | low | 6 | 0.88 | 0.5 | 1.74 | 0.08 | -0.11 | 1.86 |
|  |  |  | medium | 2 | 1.98 | 0.47 | 4.22 | 0 | 1.06 | 2.9 |
|  |  |  | high | 8 | 1.59 | 1.19 | 1.34 | 0.18 | -0.74 | 3.92 |
| seed richness | artificial |  | Low | 3 | 1.95 | 0.68 | 2.87 | 0 | 0.62 | 3.29 |
|  |  |  | medium | 9 | 2.63 | 0.84 | 3.12 | 0 | 0.98 | 4.27 |
|  | natural |  | Low | 24 | 1.95 | 0.44 | 4.38 | 0 | 1.08 | 2.82 |
|  |  |  | medium | 12 | 2.45 | 0.83 | 2.94 | 0 | 0.82 | 4.08 |
|  | semi-natural |  | Low | 4 | 1.28 | 1.06 | 1.21 | 0.23 | -0.8 | 3.35 |
|  |  |  | medium | 1 | 12.87 | 1.87 | 6.87 | 0 | 9.19 | 16.54 |
| seedling density | artificial |  | Low | 5 | 0.4 | 0.68 | 0.58 | 0.56 | -0.94 | 1.74 |
|  |  |  | medium | 5 | 1.75 | 0.82 | 2.13 | 0.03 | 0.14 | 3.37 |
|  |  |  | High | 3 | -0.14 | 0.64 | -0.22 | 0.83 | -1.4 | 1.11 |
|  | natural |  | Low | 35 | 1.22 | 0.32 | 3.82 | 0 | 0.59 | 1.84 |
|  |  |  | medium | 13 | 1.25 | 0.5 | 2.49 | 0.01 | 0.27 | 2.23 |
|  |  |  | High | 18 | 1.1 | 0.32 | 3.4 | 0 | 0.46 | 1.73 |
|  | semi-natural |  | Low | 4 | 0.5 | 0.57 | 0.87 | 0.38 | -0.62 | 1.63 |
|  |  |  | High | 6 | 1.07 | 0.48 | 2.2 | 0.03 | 0.12 | 2.02 |
| seedling richness | artificial |  | Low | 4 | 0.54 | 0.27 | 2.02 | 0.04 | 0.02 | 1.07 |
|  |  |  | medium | 3 | 0.49 | 0.34 | 1.45 | 0.15 | -0.17 | 1.15 |
|  | natural |  | Low | 6 | 0.24 | 0.19 | 1.3 | 0.19 | -0.12 | 0.61 |
|  |  |  | medium | 9 | 0.66 | 0.23 | 2.9 | 0 | 0.21 | 1.1 |
|  |  |  | High | 9 | 1.73 | 0.68 | 2.55 | 0.01 | 0.4 | 3.07 |

Table 7. Results of the three-level model for the four outcomes with matrix type as moderator. Estimates are presented as standardized mean differences Hedges’ g with their corresponding upper (UB) and lower (LB) 95% confidence intervals, and p-value (pval).

| Outcome | Matrix type | g | LB | UB | pval |
| --- | --- | --- | --- | --- | --- |
| seed density | grassland | 1.58 | 1.22 | 1.93 | 0.00 |
|  | herbaceous_open | 1.09 | 0.27 | 1.92 | 0.01 |
|  | open_cleared_land | 1.65 | 0.64 | 2.67 | 0.00 |
|  | shrubland | 0.98 | 0.64 | 1.32 | 0.00 |
|  | tree_plantation | 1.28 | -0.41 | 2.97 | 0.14 |
| seed richness | grassland | 2.55 | 1.58 | 3.52 | 0.00 |
|  | herbaceous_open | 1.19 | -0.86 | 3.24 | 0.26 |
|  | open_cleared_land | 2.76 | 1.10 | 4.42 | 0.00 |
|  | shrubland | 1.90 | 0.73 | 3.08 | 0.00 |
| seedling density | grassland | 0.99 | 0.33 | 1.65 | 0.00 |
|  | herbaceous_open | 0.70 | -1.12 | 2.53 | 0.45 |
|  | open_cleared_land | 0.99 | -0.01 | 1.98 | 0.05 |
|  | shrubland | 1.30 | 0.81 | 1.80 | 0.00 |
|  | urban_park | -0.10 | -1.82 | 1.61 | 0.90 |
| seedling richness | grassland | 1.40 | 0.81 | 1.99 | 0.00 |
|  | herbaceous_open | 0.39 | -0.92 | 1.70 | 0.56 |
|  | open_cleared_land | 0.49 | -0.62 | 1.60 | 0.39 |
|  | shrubland | 0.33 | -0.26 | 0.92 | 0.27 |

Table 8. Comparison of meta-analysis results using subgroups according to risk of bias scores for the four outcomes, with matrix type as moderator in the model. Estimates are presented as standardized mean differences Hedges’ g with their corresponding upper (UB) and lower (LB) 95% confidence intervals and other test statistics. Highlighted in green are significant effects while in orange are non-significant (p<0.05).

| **outcome** | **matrix** | **risk of bias** | **k** | **g** | **se** | **zval** | **pval** | **LB** | **UB** |
| --- | --- | --- | --- | --- | --- | --- | --- | --- | --- |
| seed density | grassland | medium | 39 | 1.31 | 0.39 | 3.38 | 0 | 0.55 | 2.07 |
|  |  | high | 32 | 1.74 | 0.17 | 10.01 | 0 | 1.4 | 2.08 |
|  | herbaceous_open | high | 6 | 1.09 | 0.31 | 3.5 | 0 | 0.48 | 1.7 |
|  | open_cleared_land | medium | 2 | 1.16 | 0.67 | 1.74 | 0.08 | -0.15 | 2.46 |
|  |  | high | 1 | 2.56 | 0.92 | 2.79 | 0.01 | 0.76 | 4.36 |
|  | shrubland | medium | 29 | 1 | 0.44 | 2.28 | 0.02 | 0.14 | 1.87 |
|  |  | high | 50 | 0.93 | 0.15 | 6.17 | 0 | 0.63 | 1.22 |
|  | tree_plantation | medium | 1 | 1.28 | 1.18 | 1.08 | 0.28 | -1.04 | 3.6 |
| seed richness | grassland | high | 9 | 5.49 | 1.48 | 3.71 | 0 | 2.59 | 8.39 |
|  |  | medium | 24 | 1.6 | 0.48 | 3.35 | 0 | 0.66 | 2.54 |
|  | herbaceous_open | high | 3 | 1.19 | 2.27 | 0.52 | 0.6 | -3.27 | 5.64 |
|  | open_cleared_land | medium | 2 | 1.99 | 0.78 | 2.55 | 0.01 | 0.46 | 3.53 |
|  | shrubland | high | 5 | 2.3 | 1.87 | 1.23 | 0.22 | -1.37 | 5.97 |
|  |  | medium | 10 | 1.6 | 0.61 | 2.62 | 0.01 | 0.4 | 2.79 |
| seedling density | grassland | high | 14 | 1.01 | 0.4 | 2.54 | 0.01 | 0.23 | 1.8 |
|  |  | medium | 3 | 0.9 | 0.79 | 1.14 | 0.25 | -0.65 | 2.46 |
|  | herbaceous_open | medium | 2 | 0.71 | 1.07 | 0.66 | 0.51 | -1.39 | 2.81 |
|  | open_cleared_land | high | 21 | 0.87 | 0.64 | 1.36 | 0.17 | -0.38 | 2.11 |
|  |  | medium | 2 | 1.26 | 1.04 | 1.21 | 0.23 | -0.78 | 3.29 |
|  | shrubland | high | 30 | 1.19 | 0.33 | 3.6 | 0 | 0.54 | 1.84 |
|  |  | medium | 14 | 1.51 | 0.47 | 3.19 | 0 | 0.58 | 2.43 |
|  | urban_park | high | 3 | -0.11 | 0.9 | -0.12 | 0.91 | -1.88 | 1.66 |
| seedling richness | grassland | high | 8 | 1.79 | 0.45 | 3.96 | 0 | 0.9 | 2.67 |
|  |  | medium | 5 | 1.11 | 0.46 | 2.41 | 0.02 | 0.21 | 2.02 |
|  | herbaceous_open | medium | 2 | 0.39 | 0.8 | 0.5 | 0.62 | -1.17 | 1.95 |
|  | open_cleared_land | medium | 3 | 0.49 | 0.69 | 0.7 | 0.48 | -0.87 | 1.85 |
|  | shrubland | high | 10 | 0.25 | 0.31 | 0.8 | 0.42 | -0.36 | 0.86 |
|  |  | medium | 3 | 0.56 | 0.73 | 0.77 | 0.44 | -0.86 | 1.99 |

Table 9. Comparison of meta-analysis results using subgroups according to risk of bias scores (RoB) for the four outcomes, with biome as moderator in the model. Estimates are presented as standardized mean differences Hedges’ g with their corresponding upper (UB) and lower (LB) 95% confidence intervals, sample size (k) and other test statistics. Highlighted in green are significant effects while in orange are non-significant (p<0.05).

| **outcome** | **biome** | **RoB** | **k** | **g** | **se** | **zval** | **pval** | **LB** | **UB** |
| --- | --- | --- | --- | --- | --- | --- | --- | --- | --- |
| seed density | Mediterranean Forests, Woodlands & Scrub | medium | 2 | 0.80 | 1.31 | 0.61 | 0.54 | -1.77 | 3.37 |
|  |  | high | 35 | 0.81 | 0.20 | 4.00 | 0.00 | 0.41 | 1.21 |
|  | Temperate Broadleaf & Mixed Forests | medium | 7 | 1.21 | 0.94 | 1.3 | 0.19 | -0.62 | 3.05 |
|  |  | high | 5 | 1.00 | 0.42 | 2.34 | 0.02 | 0.16 | 1.83 |
|  | Temperate Conifer Forests | high | 6 | 0.65 | 0.53 | 1.23 | 0.22 | -0.39 | 1.70 |
|  | Temperate Grasslands, Savannas & Shrublands | medium | 8 | 1.35 | 1.29 | 1.04 | 0.30 | -1.18 | 3.89 |
|  |  | high | 5 | 1.96 | 0.38 | 5.15 | 0.00 | 1.21 | 2.70 |
|  | Tropical & Subtropical Dry Broadleaf Forests | medium | 6 | 1.27 | 1.30 | 0.98 | 0.33 | -1.27 | 3.82 |
|  |  | high | 2 | 1.67 | 0.66 | 2.52 | 0.01 | 0.37 | 2.97 |
|  | Tropical & Subtropical Grasslands, Savannas & Shrublands | medium | 3 | 0.99 | 0.97 | 1.01 | 0.31 | -0.92 | 2.89 |
|  |  | high | 1 | 1.91 | 0.78 | 2.46 | 0.01 | 0.39 | 3.43 |
|  | Tropical & Subtropical Moist Broadleaf Forests | medium | 45 | 1.23 | 0.42 | 2.92 | 0.00 | 0.4 | 2.05 |
|  |  | high | 35 | 1.51 | 0.16 | 9.2 | 0.00 | 1.19 | 1.83 |
| seed richness | Mediterranean Forests, Woodlands & Scrub | medium | 2 | 3.20 | 0.92 | 3.47 | 0.00 | 1.39 | 5 |
|  |  | high | 1 | 13.59 | 2.08 | 6.53 | 0.00 | 9.51 | 17.67 |
|  | Temperate Broadleaf & Mixed Forests | medium | 2 | 1.83 | 0.91 | 2.02 | 0.04 | 0.06 | 3.61 |
|  | Tropical & Subtropical Grasslands, Savannas & Shrublands | high | 1 | 6.30 | 1.65 | 3.83 | 0.00 | 3.07 | 9.52 |
|  | Tropical & Subtropical Moist Broadleaf Forests | medium | 32 | 1.33 | 0.36 | 3.67 | 0.00 | 0.62 | 2.04 |
|  |  | high | 15 | 2.28 | 0.49 | 4.68 | 0.00 | 1.32 | 3.23 |
| seedling density | Mediterranean Forests, Woodlands & Scrub | medium | 2 | 2.97 | 0.64 | 4.67 | 0 | 1.72 | 4.22 |
|  |  | high | 33 | 1.31 | 0.45 | 2.88 | 0 | 0.42 | 2.2 |
|  | Montane Grasslands & Shrublands | high | 4 | 0.32 | 0.9 | 0.35 | 0.73 | -1.44 | 2.07 |
|  | Temperate Broadleaf & Mixed Forests | medium | 1 | 0.62 | 0.8 | 0.78 | 0.43 | -0.94 | 2.19 |
|  |  | high | 4 | 0.87 | 0.94 | 0.93 | 0.35 | -0.96 | 2.7 |
|  | Temperate Grasslands, Savannas & Shrublands | medium | 6 | 1.92 | 0.35 | 5.5 | 0 | 1.24 | 2.61 |
|  | Tropical & Subtropical Grasslands, Savannas & Shrublands | medium | 1 | 1.45 | 0.79 | 1.83 | 0.07 | -0.11 | 3 |
|  | Tropical & Subtropical Moist Broadleaf Forests | medium | 11 | 0.62 | 0.3 | 2.09 | 0.04 | 0.04 | 1.2 |
|  |  | high | 27 | 0.97 | 0.31 | 3.1 | 0 | 0.36 | 1.58 |
| seedling richness | Mediterranean Forests, Woodlands & Scrub | medium | 3 | 0.49 | 0.31 | 1.56 | 0.12 | -0.13 | 1.10 |
|  | Montane Grasslands & Shrublands | high | 4 | 0.20 | 1.08 | 0.18 | 0.85 | -1.91 | 2.31 |
|  | Tropical & Subtropical Grasslands, Savannas & Shrublands | medium | 1 | 2.17 | 0.58 | 3.71 | 0.00 | 1.02 | 3.31 |
|  | Tropical & Subtropical Moist Broadleaf Forests | medium | 9 | 0.62 | 0.21 | 2.90 | 0.00 | 0.20 | 1.04 |
|  |  | high | 14 | 1.03 | 0.56 | 1.83 | 0.07 | -0.07 | 2.12 |

Table 10. Results of the three-level model for the seedling outcomes with precipitation category as moderator. Precipitation category was assigned based on the annual precipitation during the study year in relation to the long-term annual rainfall for the study site from 1981-2023 using the CHIRPS dataset (Funk et al., 2015). Estimates are presented as standardized mean differences Hedges’ g with their corresponding upper (UB) and lower (LB) 95% confidence intervals, and other test statistics.

| **Outcome** | **Precipitation category** | **k** | **g** | **se** | **zval** | **pval** | **LB** | **UB** |
| --- | --- | --- | --- | --- | --- | --- | --- | --- |
| seedling density | Very dry | 14 | 1.45 | 0.57 | 2.56 | 0.01 | 0.34 | 2.56 |
|  | Dry | 19 | 1.21 | 0.38 | 3.16 | 0.00 | 0.46 | 1.96 |
|  | Normal | 6 | 1.23 | 0.60 | 2.06 | 0.04 | 0.06 | 2.41 |
|  | Wet | 19 | 0.63 | 0.32 | 1.96 | 0.05 | -0.00 | 1.27 |
|  | Very wet | 8 | 1.76 | 0.44 | 4.02 | 0.00 | 0.88 | 2.61 |
| seedling richness | Very dry | 1 | 1.10 | 0.78 | 1.40 | 0.16 | -0.44 | 2.64 |
|  | Dry | 13 | 0.96 | 0.42 | 2.30 | 0.02 | 0.14 | 1.79 |
|  | Normal | 2 | -0.02 | 0.76 | -0.03 | 0.98 | -1.51 | 1.47 |
|  | Wet | 13 | 0.58 | 0.33 | 1.77 | 0.08 | -0.06 | 1.23 |
|  | Very wet | 2 | 2.17 | 0.82 | 2.65 | 0.01 | 0.56 | 3.77 |

Table 11. Results of the multilevel meta-regression analysis with perch type, square root of the inverse of effective sample size (sqrt_inv_n_tilda) and publication year as the moderators for the four outcomes tested. The method was based on the all-in publication bias test proposed by (Nakagawa et al., 2022). Estimates are presented as standardized mean differences Hedges’ g with their corresponding upper (UB) and lower (LB) 95% confidence intervals. Significance (p<0.05) is indicated by an asterisks (*) beside the estimate.

| Outcome | Factors | g | LB | UB |  |
| --- | --- | --- | --- | --- | --- |
| Seed density | Sqrt_inv_n_tilda | 0.26 | -0.85 | 1.36 |  |
|  | Publication year | -0.01 | -0.04 | 0.02 |  |
|  | Artificial | 1.13 * | 0.48 | 1.77 |  |
|  | Natural | 1.13 * | 0.64 | 1.62 |  |
|  | Semi-natural | 1.49 * | 0.67 | 2.31 |  |
| Seed richness | Sqrt_inv_n_tilda | -3.68 * | -7.14 | -0.23 |  |
|  | Publication year | 0.09 | -0.12 | 0.29 |  |
|  | Artificial | 2.99 * | 0.89 | 5.1 |  |
|  | Natural | 2.93 * | 1.02 | 4.85 |  |
|  | Semi-natural | 10.73 * | 7.39 | 14.08 |  |
| Seed richness (w/o outlier study 49) | | Sqrt_inv_n_tilda | -1.48 | -4.57 | 1.61 |
|  |  | Publication year | 0.13 * | 0.03 | 0.23 |
|  |  | Artificial | 2.53 * | 0.98 | 4.09 |
|  |  | Natural | 2.62 * | 1.29 | 3.95 |
|  |  | Semi-natural | 1.05 | -1.77 | 3.86 |
| Seedling density | Sqrt_inv_n_tilda | -0.16 | -2.23 | 1.91 |  |
|  | Publication year | 0 | -0.05 | 0.05 |  |
|  | Artificial | 0.85 | -0.44 | 2.14 |  |
|  | Natural | 1.29 * | 0.36 | 2.22 |  |
|  | Semi-natural | 0.86 | -0.46 | 2.18 |  |
| Seedling richness | Sqrt_inv_n_tilda | 1.75 | -1.4 | 4.9 |  |
|  | Publication year | 0.01 | -0.06 | 0.07 |  |
|  | Artificial | -0.05 | -1.59 | 1.48 |  |
|  | Natural | 0.17 | -1.24 | 1.58 |  |

Table 12. Results of the multilevel meta-regression analysis without modifiers using dataset with and without outliers. Outliers were identified as such if the study’s confidence interval overlapped with the confidence interval of the pooled effect (Harrer et al., 2019). Estimates are presented as standardized mean differences Hedges’ g with their sample size (k), upper (UB) and lower (LB) 95% confidence intervals. Asterisks beside the estimate indicates significant effect (p<0.05). No significant change in the effect estimates was observed with the removal of the outliers.

| **Outcome** | **k** | | **g** | | **LB** | | **UB** |  |
| --- | --- | --- | --- | --- | --- | --- | --- | --- |
| Without outliers |  | |  | |  | |  |  |
| seed density | 126 | | 1.03 * | | 0.94 | | 1.12 |  |
| seed richness | 40 | | 2.12 * | | 1.62 | | 2.62 |  |
| seedling density | 74 | | 0.98 * | | 0.80 | | 1.16 |  |
| seedling richness | 27 | | 0.56 * | | 0.26 | | 0.86 |  |
| With outliers |  | |  | |  | |  |  |
| seed density | 160 | 1.26 * | | 1.02 | | 1.5 | | |
| seed richness | 53 | 2.16 * | | 1.48 | | 2.84 | | |
| seedling density | 89 | 1.09 * | | 0.75 | | 1.44 | | |
| seedling richness | 31 | 0.8 * | | 0.36 | | 1.25 | | |

Table 13. Results of the multilevel meta-regression analysis according to perch type using dataset with and without outliers. Outliers were identified as such if the study’s confidence interval overlapped with the confidence interval of the pooled effect (Harrer et al., 2019). Estimates are presented as standardized mean differences Hedges’ g with their sample size (k), upper (UB) and lower (LB) 95% confidence intervals, and p-value. Asterisks beside the estimate indicates significant effect (p-val<0.05). Highlights in peach indicate changes in the significance of the effect with the removal of the outliers.

| **Outcome** | **Perch Type** | **k** | **g** | **LB** | **UB** | **p-val** |
| --- | --- | --- | --- | --- | --- | --- |
| **WITH OUTLIERS** |  |  |  |  |  |  |
| seed density | Artificial | 160 | 1.21 * | 0.77 | 1.65 | 0 |
|  | Natural | 160 | 1.22 * | 0.94 | 1.51 | 0 |
|  | Semi-natural | 160 | 1.59 * | 0.98 | 2.21 | 0 |
|  |  |  |  |  |  |  |
| seed richness | Artificial | 53 | 1.97 * | 0.51 | 3.43 | 0.01 |
|  | Natural | 53 | 1.68 * | 0.38 | 2.97 | 0.01 |
|  | Semi-natural | 53 | 8.6 * | 5.75 | 11.44 | 0 |
|  |  |  |  |  |  |  |
| seedling density | Artificial | 89 | 0.8 | -0.02 | 1.62 | 0.06 |
|  | Natural | 89 | 1.22 * | 0.79 | 1.64 | 0 |
|  | Semi-natural | 89 | 0.81 | -0.05 | 1.68 | 0.06 |
|  |  |  |  |  |  |  |
| seedling richness | Artificial | 31 | 0.52 | -0.52 | 1.55 | 0.33 |
|  | Natural | 31 | 0.89 * | 0.36 | 1.41 | 0 |
|  |  |  |  |  |  |  |
| **WITHOUT OUTLIERS** | |  |  |  |  |  |
| seed density | Artificial | 126 | 1.22 * | 1.01 | 1.42 | 0 |
|  | Natural | 126 | 0.97 * | 0.86 | 1.07 | 0 |
|  | Semi-natural | 126 | 1.09 * | 0.81 | 1.36 | 0 |
|  |  |  |  |  |  |  |
| seed richness | Artificial | 40 | 2.24 * | 1.46 | 3.03 | 0 |
|  | Natural | 40 | 2.16 * | 1.49 | 2.84 | 0 |
|  | Semi-natural | 40 | 1.28 | -0.56 | 3.12 | 0.17 |
|  |  |  |  |  |  |  |
| seedling density | Artificial | 74 | 0.9 * | 0.48 | 1.33 | 0 |
|  | Natural | 74 | 0.95 * | 0.74 | 1.16 | 0 |
|  | Semi-natural | 74 | 1.24 * | 0.76 | 1.71 | 0 |
|  |  |  |  |  |  |  |
| seedling richness | Artificial | 27 | 0.52 | -0.2 | 1.24 | 0.16 |
|  | Natural | 27 | 0.6 * | 0.2 | 1 | 0 |

Table 14. Comparison of the multilevel meta-regression analysis for seedling density with and without variance imputation. Eight studies were excluded from the original data frame as they have zero means and variances, but these were imputed with variances of 0.1, 1, and 10 for both control and intervention groups for sensitivity analysis. The pooled effect estimate remained positive despite inclusion of the eight studies. Estimates are presented as standardized mean differences Hedges’ g with their sample size (k), upper (UB) and lower (LB) 95% confidence intervals.

| Dataset | k | g | LB | UB |
| --- | --- | --- | --- | --- |
| **Original (excluded zero data studies)** |  |  |  |  |
| Artificial | 13 | 0.8 | -0.02 | 1.62 |
| Natural | 66 | 1.22 * | 0.79 | 1.64 |
| Semi-natural | 10 | 0.81 | -0.05 | 1.68 |
| **Imputed variance of 0.1** |  |  |  |  |
| Artificial | 15 | 0.68 | -0.08 | 1.44 |
| Natural | 70 | 1.21 * | 0.78 | 1.63 |
| Semi-natural | 12 | 0.73 | -0.12 | 1.57 |
| **Imputed variance of 1.0** |  |  |  |  |
| Artificial | 15 | 0.68 | -0.08 | 1.44 |
| Natural | 70 | 1.21 * | 0.78 | 1.63 |
| Semi-natural | 12 | 0.73 | -0.12 | 1.57 |
| **Imputed variance of 10** |  |  |  |  |
| Artificial | 15 | 0.68 | -0.08 | 1.44 |
| Natural | 70 | 1.21 * | 0.78 | 1.63 |
| Semi-natural | 12 | 0.73 | -0.12 | 1.57 |


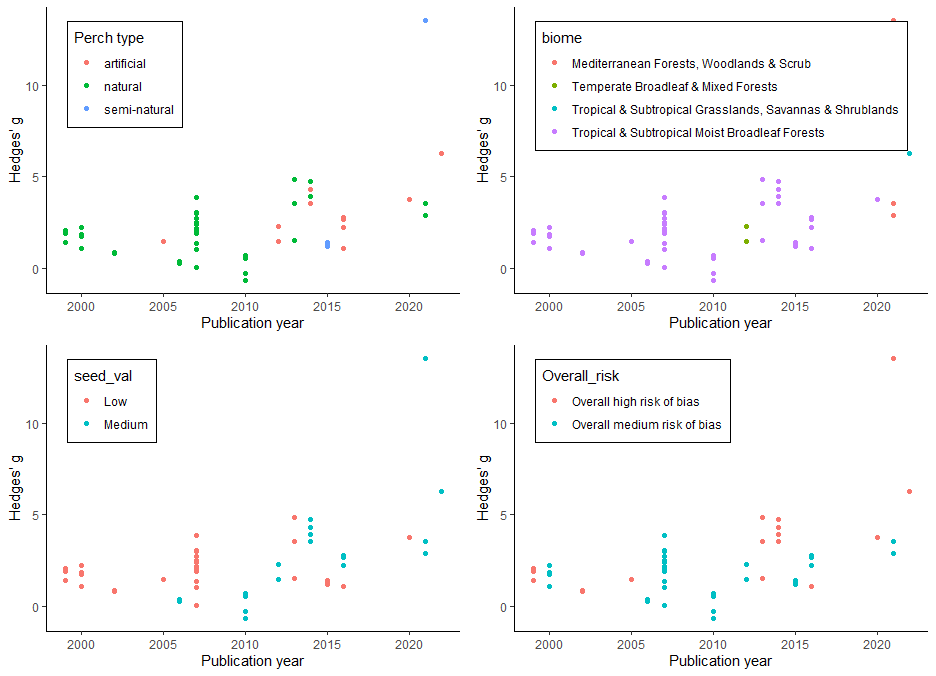


Figure 2. Scatterplot of the effect estimates on seed richness over publication year. Points are coloured based on perch type (top left), biome (top right), method validity score (bottom left), and overall risk of bias (bottom right).

Figure 3. Funnel plots of meta-analytic fixed effects models without moderators for each of the four outcomes. Points correspond to studies, which are coloured according to the overall risk of bias: red for high risk and orange for medium risk.
